# Seeding with minimized subsequence

**DOI:** 10.1093/bioinformatics/btad218

**Published:** 2023-06-30

**Authors:** Xiang Li, Qian Shi, Ke Chen, Mingfu Shao

**Affiliations:** Department of Computer Science and Engineering, The Pennsylvania State University, University Park, PA 16802, USA; Department of Computer Science and Engineering, The Pennsylvania State University, University Park, PA 16802, USA; Department of Computer Science and Engineering, The Pennsylvania State University, University Park, PA 16802, USA; Department of Computer Science and Engineering, The Pennsylvania State University, University Park, PA 16802, USA; Huck Institutes of the Life Sciences, The Pennsylvania State University, University Park, PA 16802, USA

## Abstract

**Motivation:**

Modern methods for computation-intensive tasks in sequence analysis (e.g. read mapping, sequence alignment, genome assembly, etc.) often first transform each sequence into a list of short, regular-length seeds so that compact data structures and efficient algorithms can be employed to handle the ever-growing large-scale data. Seeding methods using *k*mers (substrings of length *k*) have gained tremendous success in processing sequencing data with low mutation/error rates. However, they are much less effective for sequencing data with high error rates as *k*mers cannot tolerate errors.

**Results:**

We propose SubseqHash, a strategy that uses subsequences, rather than substrings, as seeds. Formally, SubseqHash maps a string of length *n* to its smallest subsequence of length *k*, *k *<* n*, according to a given order overall length-*k* strings. Finding the smallest subsequence of a string by enumeration is impractical as the number of subsequences grows exponentially. To overcome this barrier, we propose a novel algorithmic framework that consists of a specifically designed order (termed ABC order) and an algorithm that computes the minimized subsequence under an ABC order in polynomial time. We first show that the ABC order exhibits the desired property and the probability of hash collision using the ABC order is close to the Jaccard index. We then show that SubseqHash overwhelmingly outperforms the substring-based seeding methods in producing high-quality seed-matches for three critical applications: read mapping, sequence alignment, and overlap detection. SubseqHash presents a major algorithmic breakthrough for tackling the high error rates and we expect it to be widely adapted for long-reads analysis.

**Availability and implementation:**

SubseqHash is freely available at https://github.com/Shao-Group/subseqhash.

## 1 Introduction

Transforming a sequence into a list of seeds (also known as markers) that are then processed in place of the original sequence is a common approach in sequence analysis as a remedy for the dreadfully expensive full-length comparisons. The resulting seeds of such a transformation are often regular in length and much shorter than the original sequences, making it possible to apply efficient data structures and fast algorithms. For example, in read mapping, the popular seed-and-extend strategy (Altschul et al. 1990, 1997) first identifies seed-matches (i.e. pairs of identical seeds; also known as anchors) between a read and a reference, then performs local alignment around these seeds to look for statistically significant matches. In sequence alignment, seed-matches across sequences are first identified, followed by efficient chaining algorithms to find a co-linear chain of matching seeds that maximizes a scoring function (Myers and Miller 1995; Abouelhoda and Ohlebusch 2005; Jain et al. 2022). In genome assembly, a (sparse) de Bruijn graph can be constructed in which seeds are used as vertices and two seeds are linked by an edge if they are adjacent in some reads (Lin et al. 2016; Ruan and Li 2020; Ekim et al. 2021; Rautiainen and Marschall 2021; Bankevich et al. 2022). To mitigate all-versus-all pairwise comparisons in applications, such as aligning multiple sequences and constructing overlap/string graphs (Koren et al. 2017; Nurk et al. 2020; Cheng et al. 2021), seeds can be used to bucket sequences (i.e. assigning a sequence into buckets labeled by its own seeds), followed by pairwise comparisons in individual buckets (Berlin et al. 2015; Song et al. 2020). The efficiency and accuracy of these methods heavily rely on the quality of the generated seeds. Desired properties include high sensitivity (i.e. biologically related sequences producing many seed-matches) and a low false positive rate (i.e. unrelated sequences producing few seed-matches).

Arguably the most widely used seeds are simply *k*mers (i.e. substrings of fixed length *k*). Sketching approaches, such as Minimizers (Schleimer et al. 2003; Roberts et al. 2004a,b; Marçais et al. 2018) or syncmers (Edgar 2021), are often combined to select a subset of *k*mers aiming for scaling. Seeding methods using *k*mers have gained success in almost all aspects of sequence analysis especially on data with low error rates. However, requiring exact matches of *k* consecutive characters becomes less effective in comparing biologically related sequences with high mutation rates or error rates. Such scenarios include comparing homologous genes or whole genomes from distant species and processing long-read sequencing data generated by PacBio (Rhoads and Au 2015) and Oxford Nanopore (Jain et al. 2018) technologies. Observe that a single mutation/error can change *k* consecutive *k*mers in the sequence and the probability of a *k*mer remaining intact under a uniform mutation model decreases exponentially as *k* grows (Blanca et al. 2022). This greatly challenges the *k*mer-based seeding methods and puts them in a dilemma: choosing a large *k* results in few seed-matches even in biologically related sequences (i.e. low sensitivity), while making *k* too small suffers from a high false positive rate as unrelated sequences can, by chance, share many short common substrings. Existing tools, such as KmerGenie (Chikhi and Medvedev 2014), help select a size of *k*mers that balances its sensitivity and false positive rate based on the data. But due to the intrinsic weakness of *k*mers against mutations, even an optimal choice of *k* can still produce unsatisfactory results.

Alternative approaches have been proposed to collect non-consecutive characters as seeds. Spaced seeds (Califano and Rigoutsos 1993; Ma et al. 2002) are extracted by applying a predefined pattern, such as 1110111 on a string where a 1 means the character at that position is taken and a 0 means it is ignored/masked. The masked positions allow seed-matches to occur over substitutions, but because of the fixed pattern, spaced seeds can only handle substitutions at predefined locations and are still vulnerable to insertions and deletions (indels). Indel seeds (Mak et al. 2006) use patterns with wild-cards to accommodate certain numbers of indels. But again only indels at the predefined regions can be managed. Using multiple patterns (Li et al. 2004; Kucherov et al. 2005; Sun and Buhler 2005; Farach-Colton et al. 2007) alleviates the restrictions of a single pattern at the cost of more computations but still cannot handle mutations/errors at arbitrary locations.

It is also worth mentioning that there have been successful seeding methods that combine two or more pre-extracted (shorter) seeds. For example, grouped methods (Du et al. 2019) use two or more independently produced *k*mers as seeds. The Order Min Hash approach (Marçais et al. 2019) selects multiple *k*mers from a sequence with relative positions preserved. The recently proposed *k*mer-alternative method strobemer and its variants (Sahlin 2021; Maier and Sahlin 2022; Sahlin 2022) pick and concatenate *k*mers from multiple consecutive predetermined windows. This provides more flexibility on the spacing between extracted *k*mers and therefore is less susceptible to different mutation rates. Nonetheless, since these methods all use *k*mers as building blocks, they cannot fully resolve the drawback of *k*mer-based methods. In addition, we consider these approaches orthogonal to the “basic” seeding methods (such as *k*mers and our method described below) in the sense that they can be applied on top of any kind of basic seeds.

In this article, we explore the use of subsequences, rather than substrings, as seeds. The key observation is that two similar strings may share few or even zero substrings (of length *k*) but can contain many common subsequences (of length *k* or longer). Consider an example with two similar strings of length 7: s=ACGCCTA and t=ACGGCTA that differ by one substitution in the middle (i.e. their edit distance is 1). Clearly, ***s*** and ***t*** do not share any *k*mer for k≥4, leading to zero seed-matches for any *k*mer-based seeding methods (when k≥4 is used). On the other hand, two-thirds of the total 21 unique length-4 subsequences of ***s*** are also subsequences of ***t***. In fact, the Jaccard index between the two sets of length-4 subsequences of ***s*** and ***t*** is 14/(21+24−14)≈0.45. According to the property of MinHash (Broder 1997), if the “smallest” subsequences of length 4 (with respect to a fully random order, i.e. an order picked uniformly at random from all possible orders over strings of length 4) from ***s*** and ***t*** are picked as their respective seeds, then the probability of hash collision (i.e. producing a seed-match) is also about 0.45. Although rather simple, this example demonstrates the potential advantage of using subsequences as seeds: as it is more tolerant to edits happening at any position, it is more likely to produce seed-matches for similar sequences, and therefore provides enhanced sensitivity, particularly for data with a high mutation/error rate.

To this end, we present a subsequence-based seeding method named SubseqHash. It maps a string of length *n* to its smallest subsequence of length *k* (i.e. the seed), *k *<* n*, according to a given order overall length-*k* strings. There is one caveat: the number of subsequences grows exponentially in a string, which makes the computation of the smallest subsequence intractable when a fully random order is used. To overcome this difficulty, we propose a new algorithmic framework, consisting of a specifically designed order, named ABC order, and an algorithm that finds the smallest subsequence under an ABC order in polynomial time (Section 2). We show experimentally that an ABC order exhibits similar properties as a fully random order and that the probability of hash collision is close to that when a fully random order is used (Sections 3.1 and 3.2). We finally demonstrate the superiority of SubseqHash over the *k*mer-based seeding method Minimizer in several applications, including sequence alignment, read mapping, and overlap detection (Sections 3.4–3.6).

## 2 SubseqHash

### 2.1 Definitions of SubseqHash

Let ***x*** be a string of length *n* over an alphabet Σ. Given an integer k≤n, denote by $S_{k}(x)$ the set of all subsequences of ***x*** of length *k*. Let *π* be a permutation of $\Sigma^{k}$, i.e. *π* defines an order over all possible strings of length *k*. Define $h_{\pi }(x)$ as the smallest string in $S_{k}(x)$ according to *π*. In other words, the function $h_{\pi }$ maps a string of length *n* to its smallest (defined by *π*) subsequence of length *k*; formally, $h_{\pi }(x)=\arg\min_{z\in S_{k}(x)}\pi (z)$, where π(z) is the rank of ***z*** in the order defined by *π*. We use SubseqHash to term such a hashing function $h_{\pi }$.

### 2.2 Probability of hash collision

The intuition behind SubseqHash is that a few edits between two sequences may destroy most of their common substrings but many common subsequences can survive. We use the Jaccard index to measure the similarity of two sets. Given two strings ***x*** and ***y***, the Jaccard index for their subsequences of length *k* is defined as $J(x,y):=|S_{k}(x)\cap S_{k}(y)|/|S_{k}(x)\cup S_{k}(y)|$. The Jaccard index for their substrings can be defined similarly. In Supplementary Notes S1 and S2, we estimate and compare the Jaccard index for subsequences and substrings; in general, the Jaccard index for subsequences is larger than that for substrings, verifying the intuition.

We say an order *π* over $\Sigma^{k}$ is “fully random” if *π* is drawn uniformly at random from all orders. For a fully random order *π*, the probability of hash collision of $h_{\pi }$ is exactly the Jaccard index for subsequences according to the property of MinHash (Broder 1997), i.e. $\mathrm{Pr}\left(h_{\pi }(x)=h_{\pi }(y)\right)=J(x,y)$ for any two strings ***x*** and ***y***. If *π* is not fully random then this may not hold. For example, when the lexicographic order is used, the empirical probability of hash collision reduces considerably (Section 3.2).

### 2.3 Algorithmic framework for constructing SubseqHash

The complexity of calculating $h_{\pi }(x)$ for a given string ***x*** also depends on the choice of *π*. For a fully random order *π*, one can compute $h_{\pi }(x)$ by enumerating all subsequences of ***x*** and picking the smallest one. Another approach is to traverse all strings of length *k* down the order *π* and return the first one that is a subsequence of ***x***. (Determining if a string ***z*** is a subsequence of another string ***x*** can be done in O(|z|+|x|) time, where |·| denotes the length of a string.) Both approaches run in exponential time. On the other hand, when the lexicographic order *π* is used, $h_{\pi }(x)$ can be computed in linear time; the downside is that the probability of hash collision gets reduced significantly as stated above.

We propose a novel approach to balance performance and efficiency. The idea is to use a special order *π* that allows for computing $h_{\pi }(x)$ in polynomial time. Such a special order is not fully random, but is designed to be “quite” random, and therefore achieves a probability of hash collision comparable with a fully random order. The special order *π* and the polynomial time algorithm are described in the next two sections.

### 2.4 The ABC order

We start with designing an order *π* over $\Sigma^{k}$, named ABC order. Let d≥1 be an integer parameter. Essentially, *π* is given as a scoring function that maps a string $z\in \Sigma^{k}$ to a pair π(z):=(ψ(z),ω(z)), where ψ(z)∈{0,1,…,d−1} and ω(z)∈R. Then all strings in $\Sigma^{k}$ are ordered as follows: for $z,z\prime \in \Sigma^{k}$, define π(z)<π(z′) if and only if ψ(z)<ψ(z′), or ψ(z)=ψ(z′) and |ω(z)|>|ω(z′)|. The construction of π(·) (see below) makes it extremely unlikely to have ψ(z)=ψ(z′) and |ω(z)|=|ω(z′)| for z≠z′. When such a rare case happens, we define π(z)<π(z′) if and only if ***z*** is lexicographically smaller than z′.

We specify such a function *π* for DNA strings by assuming Σ={A=1, C=2, G=3, T=4}. The function *π* is governed by three (random) tables *A*, *B*, and *C*; hence the name. Table *A* is a 3D real matrix of dimension k×d×|Σ|, i.e. $A\in R^{k\times d\times |\Sigma |}$. Table *B* is also of dimension k×d×|Σ|, where B[i][j][σ]∈{(+1,+1),(+1,−1),(−1,+1),(−1,−1)}, 1≤i≤k, 0≤j≤d−1, and σ∈Σ. Table *C* has dimension k×|Σ|, where C[i][σ]∈{0,1,…,d−1}, 1≤i≤k and σ∈Σ.

These three tables are randomly generated in the following way. Each element A[i][j][σ] is drawn independently and uniformly at random from a predetermined subset of R; our implementation uses $\left[2^{30},2^{31}\right]$. For any fixed *i* and *j*, 1≤i≤k and 0≤j≤d−1, B[i][j][σ] is picked from the four pairs {(+1,+1),(+1,−1),(−1,+1),(−1,−1)} uniformly at random without replacement, i.e. $B[i][j][\sigma_{1}]\neq B[i][j][\sigma_{2}]$ if $\sigma_{1}\neq \sigma_{2}$. Last, for any fixed *i*, 1≤i≤k, each element C[i][σ] is drawn independently and uniformly at random from {0,1,2,…,d−1}. If the parameter d≥|Σ|=4, then we do this without replacement, i.e. $C[i][\sigma_{1}]\neq C[i][\sigma_{2}]$ if $\sigma_{1}\neq \sigma_{2}$. Please see Supplementary Note S3 for an example.

Once the tables *A*, *B*, and *C* are generated, the scoring function π(z)=(ψ(z),ω(z)) is determined for any string $z\in \Sigma^{k}$. Write $z=z_{1}z_{2}\cdots z_{k}$, where $z_{i}\in \Sigma ,\;1\leq i\leq k$. For the sake of simplicity, denote $\psi (z_{1}z_{2}\cdots z_{i})$ and $\omega (z_{1}z_{2}\cdots z_{i})$ by *ψ_i_* and *ω_i_*, respectively; also denote the first and the second element in the pair B[i][j][σ] by $B[i][j]{\left[\sigma \right]}_{1}$ and $B[i][j]{\left[\sigma \right]}_{2}$, respectively.

The initial values are set to $\psi_{0}=0$ and $\omega_{0}=0$. For 1≤i≤k, we use recurrences and


$$\psi_{i}=(\psi_{i-1}+C[i][z_{i}])\;\mathrm{mod}\;d,$$

$$\omega_{i}=\omega_{i-1}\cdot B[i][\psi_{i}]{\left[z_{i}\right]}_{1}+A[i][\psi_{i}][z_{i}]\cdot B[i][\psi_{i}]{\left[z_{i}\right]}_{2}.$$

Observe that if d≥2, then one edit in the string is guaranteed to alter the value of *ψ*, while two edits have a small chance (approximately 1/d) to result in the same *ψ*. In addition, due to the use of –1 in table *B*, the value of *ω* can be substantially changed with even a single mutation. Combined, a few edits can cause a drastic change in both *ψ* and *ω*, and therefore the rank of strings in the order, which is a desired property; see Section 3.1 for more discussions.

### 2.5 Algorithm for computing the smallest subsequence with an ABC order

Let *π* be an ABC order. We design an efficient algorithm to find $h_{\pi }(x)=\arg\min_{z\in S_{k}(x)}\pi (z)$ for any given $x=x_{1}x_{2}\cdots x_{n}\in \Sigma^{n}$. Since the scoring function π(z) is defined by recurrences, it is natural to solve $h_{\pi }(x)$ using a dynamic programming algorithm. Consider a subproblem parameterized by *l*, *i*, and *j*, where 1≤l≤n, 1≤i≤k, and 0≤j<d, which is defined to seek a subsequence $z_{1}z_{2}\cdots z_{i}$ of $x_{1}x_{2}\cdots x_{l}$ such that $\psi (z_{1}z_{2}\cdots z_{i})=j$ and $\omega (z_{1}z_{2}\cdots z_{i})$ is minimized or maximized. We calculate both the maximized and minimized values because they would be switched when encountering a pair from table *B* with –1 being its first element. Furthermore, both values are needed at the end since the definition of an ABC order requires finding the subsequence that maximizes the absolute value of ω(·).

Formally, for each *l*, *i*, and *j*, we define subproblems: and


$$T_{\min}[l][i][j]:=\underset{z\in S_{i}(x_{1}x_{2}\cdots x_{l})\;\;\text{and}\;\;\psi (z)=j}{\min}\omega (z)$$

$$T_{\max}[l][i][j]:=\underset{z\in S_{i}(x_{1}x_{2}\cdots x_{l})\;\;\text{and}\;\;\psi (z)=j}{\max}\omega (z).$$

They can be calculated with the recurrences below, in which $j\prime =(j-C[i][x_{l}]+d)\;\mathrm{mod}\;d$.


$$\begin{array}{c} T_{\min}[l][i][j]=\min\{\begin{array}{c} T_{\min}[l-1][i][j]\\ A[i][j][x_{l}]\cdot B[i][j]{\left[x_{l}\right]}_{2}+\{\begin{array}{c} +T_{\min}[l-1][i-1][j\prime ]\\ \left(\text{if}\;\;B[i][j]{\left[x_{l}\right]}_{1}=+1\right)\\ -T_{\max}[l-1][i-1][j\prime ]\\ \left(\text{if}\;\;B[i][j]{\left[x_{l}\right]}_{1}=-1\right) \end{array} \end{array} \end{array}$$

$$\begin{array}{c} T_{\max}[l][i][j]=\max\{\begin{array}{c} T_{\max}[l-1][i][j]\\ A[i][j][x_{l}]\cdot B[i][j]{\left[x_{l}\right]}_{2}+\{\begin{array}{c} +T_{\max}[l-1][i-1][j\prime ]\\ \left(\text{if}\;\;B[i][j]{\left[x_{l}\right]}_{1}=+1\right)\\ -T_{\min}[l-1][i-1][j\prime ]\\ \left(\text{if}\;\;B[i][j]{\left[x_{l}\right]}_{1}=-1\right) \end{array} \end{array} \end{array}.$$

Initially, for any $0\leq l\leq n,\;T_{\min}[l][0][0]=T_{\max}[l][0][0]=0$ and $T_{\min}[l][0][j]=T_{\max}[l][0][j]=\text{NaN}$ if j≠0. Tables *T*_min_ and *T*_max_ can then be filled using the above recurrences. Subsequently, for 0≤j<d, we record the values


$$T[n][k][j]:=\max\{\left|T_{\min}[n][k][j]\right|,\left|T_{\max}[n][k][j]\right|\}.$$

In these processes, if any of the three arithmetic operations {+,−,|·|} involves NaN as an operand, then the result is also an NaN. The min and max operations ignore NaN and only work on numerical operands, unless there is none, in which case an NaN is returned. At the end, we calculate


$$\psi_{opt}=\min\{j|T[n][k][j]\neq \text{NaN}\}\;\;\text{and}\;\;\omega_{opt}=T[n][k][\psi_{opt}].$$

The optimal subsequence $h_{\pi }(x)$, i.e., the subsequence *z* of *x* with $\pi (z)=(\psi_{opt},\;\omega_{opt}),\;$can be obtained by traceback. The entire algorithm runs in *O*(*nkd*) time.

### 2.6 Using SubseqHash in practice

It is desirable for a seeding/hashing function to be “locality-sensitive,” i.e. the probability of hash collision for a pair of strings ***x*** and ***y*** is high if they are similar (say, measured with the edit distance), and at the same time such probability becomes low if ***x*** and ***y*** are not similar. These desired properties can also be interpreted as having high sensitivity (more true seed-matches) and a low false positive rate (fewer false seed-matches). For SubseqHash coupled with an ABC order, the choice of *n* and *k* balances these two measures. Generally speaking, a larger *n* lowers the false positive rate while a smaller *n* provides higher sensitivity; for a fixed *n*, increasing the difference between *n* and *k* improves sensitivity while decreasing the difference reduces the number of false seed-matches.

Again, two similar strings ***x*** and ***y*** are likely to share some (long) subsequences. In fact, assume the edit distance between two length-*n* strings ***x*** and ***y*** is *e*_1_, then a shared subsequence of length *k* is guaranteed if $k\leq n-e_{1}$. In this case, the probability of hash collision under SubseqHash between ***x*** and ***y*** is strictly positive. However, the probability might be small so that one round of SubseqHash may not actually pick a common subsequence of the two strings (see Section 3.2 for some experimental results). To boost the chance of getting a seed-match, one can “repeat” SubseqHash several times independently, each of which uses a different set of random A/B/C tables. Assume that *p* is the probability of hash collision of calling SubseqHash once, then with *t* repeats, the probability of having at least one seed-match is $1-{\left(1-p\right)}^{t}$. Repeats may also increase the false positive seed-matches, but it can be well controlled by picking a large *n* and a *k* that is close to *n*. Specifically, two dissimilar strings (i.e. their edit distance *e*_2_ is large) of length *n* will not share any subsequence of length *k* if $k>n-e_{2}/2$. In Sections 3.4–3.6, we show that repeats can boost sensitivity while maintaining a low false positive rate (i.e. high precision).

On the other hand, repeats are not as practical for substring-based seeding methods. This is because it is easy for two similar strings not to share any substring of a reasonable length. In fact, a shared substring of length *k* is guaranteed only if $k\leq n-ke_{1}$, as one edit can break up to *k* substrings of length *k*. When two (similar) strings do not share any length-*k* substring, a seed-match will not be produced regardless of the number of repeats. In the experiments, we include the comparison with “all-kmers” (i.e. every sliding window of length *k* in a sequence is collected as a seed), which is the limit of repeating Minimizers.

## 3 Results

### 3.1 Comparison of orders

We propose a measure to characterize the similarity of neighboring strings in an order. Observe that, the neighboring strings in the lexicographic order are similar, while they are independent and therefore distant from each other in a fully random order. Let *O* be an order over $\Sigma^{k}$ and let O[i] be its *i*-th string. We define the 2*w* strings in a window centered at O[i] as the neighboring strings of O[i], where *w* is a parameter. We use $D_{w}(O,i):=\min_{i-w\leq j\leq i+w,j\neq i}e\mathrm{dit}(O[j],O[i])$ to quantify how similar O[i] is with its neighboring strings, where edit(·,·) denotes the edit distance. We finally calculate the “averaged minimum neighboring edit distance” (AMNED) over the *m* smallest (top-ranked) strings in the order, i.e. $D_{w,m}(O):=\sum_{i=1}^{m}D_{w}(O,i)/m$. We consider top-ranked strings in an order as they are more likely to be hashed to in SubseqHash.

We compare the AMNEDs of ABC orderings, fully random orders, and the lexicographic order for strings of length *k *=* *15. To generate the top *m* strings in an ABC order, we randomly generate tables *A*, *B*, and *C* (with three choices of *d *=* *1, 11, 31), calculate the score (ψ(·),ω(·)) of all strings in $\Sigma^{k}$, sort them, and pick the top *m* strings. The top *m* strings of a fully random order are generated by independently simulating random strings of length *k* until *m* distinct ones are available. Note that the AMNED is 1 for the lexicographic order regardless the choice of *w* and *m*.

**Figure 1.**
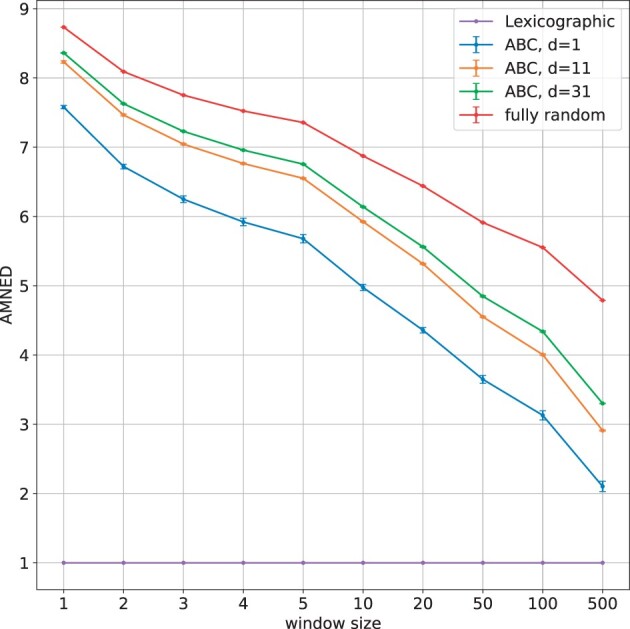
The AMNED of different orders over strings of length *k *=* *15 evaluated with varying *w* (*x*-axis) and *m *=* *10 000. The point and error bar show the mean and standard deviation over 10 individual runs. Results for different values of *m* are available in Supplementary Fig. S3.

Figure 1 and Supplementary Fig. S3 report the averaged AMNEDs and the standard deviation for the above three orders (10 repetitions for the ABC order and the fully random order) using different choices of *w* and *m*. The average AMNED for the ABC order is reasonably large, suggesting that the ABC order is “quite” random, in the sense that nearby strings are dissimilar. There is still a gap between an ABC order and a fully random order, but the gap is gradually decreased as *m* grows. Changing *d* from 1 to 11 for an ABC order significantly increases the AMNED, suggesting the effectiveness of using table *C*. There is a small growth when *d* is further increased to 31; we therefore pick *d *=* *11 in the experimental studies.

### 3.2 Comparison of probability of hash collision

We compare the probability of hash collision achieved by Minimizer and SubseqHash. Each seeding method takes a pair of strings (x,y) as input, and extracts a single seed from each string. For Minimizer, the seed of ***x*** is the smallest *k*mer among the (|x|−k+1)*k*mers in ***x*** according to a fully random order. For SubseqHash, the seed of ***x*** is the smallest subsequence of length *k* in ***x***, according to the chosen order. For the lexicographic order, linear algorithm exists to find the optimal seed; for the ABC order, algorithm in Section 2.5 is used; for a fully random order, we use a brute-force approach to find the smallest seed (and hence we are not able to report the results for large *k* in Supplementary Fig. S5). A seed of ***y*** will be extracted independently but with the shared order used for ***x***. We then check if the two seeds are identical (i.e. a hash collision).

We use simulations to estimate the probability of hash collisions. To simulate pairs of strings, we start with ***x*** being a random string of length *n*, *n *=* *20 or *n *=* *30. We then apply *n* random evolutionary events sequentially on ***x***, with each event with probability of 1/3 being a substitution, a deletion, or an insertion. We make sure that each position can be only mutated once. We collect both the intermediate *n−*1 strings and the final string, resulting in *n* pairs of strings $(x,y_{i}),\;i=1,2,\ldots ,n$. All pairs are categorized according to the edit distance, i.e. pair $\left(x,y_{i}\right)$ is put into the *j*-th category if $\mathrm{edit}(x,y_{i})=j$. Note that there are *i* mutations simulated from ***x*** to $y_{i}$, but it is not necessarily true that $\mathrm{edit}(x,y_{i})=i$. Notice also that it is possible that $\left|y_{i}|\neq |x\right|$, but a seed of the same length (a *k*mer for Minimizer and a subsequence of length *k* for SubseqHash) will be extracted from them.

**Figure 2.**
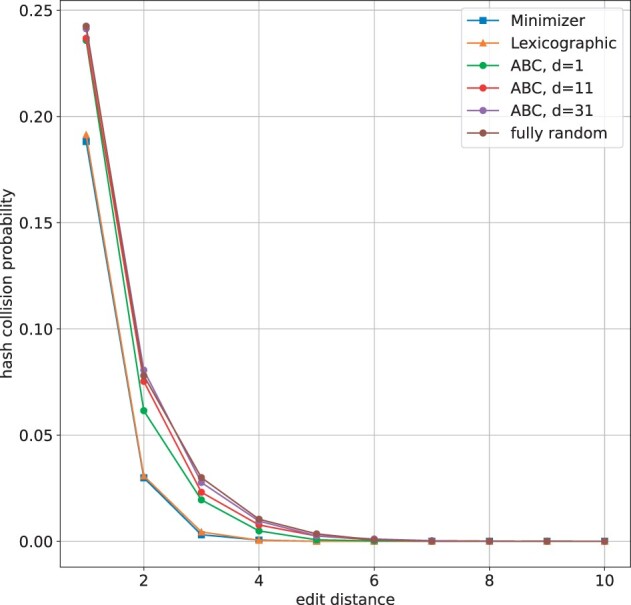
The probability of hash collision estimated, using simulations, for different seeding methods with *n *=* *20 and *k *=* *16. More results with different *n* and *k* are available in Supplementary Figs S4 and S5.

We simulate 10 000 pairs of strings following above procedure, and in each of the 10 categories where edit distance is from 1 to 10, we calculate the frequency of hash collisions and use it as an estimation of the probability of hash collision. The results are shown in Fig. 2 and Supplementary Figs S4 and S5. Minimizer and SubseqHash are compared when extracting seeds of the same length (the same *k*). Observe that in all settings SubseqHash coupled with ABC order achieves much higher probability than Minimizer when the edit distance is in a range of 1–5, indicating the superiority of SubseqHash over Minimizer in hashing similar strings but with high error rates. The probability of hash collision of SubseqHash coupled with the lexicographic order is very similar to that of the Minimizer, suggesting the necessity of a more random order (than lexicographic order) to make SubseqHash more sensitive. The probability of hash collision of SubseqHash gets much improved when *d *=* *11 is used in ABC order than *d *=* *1, again indicating the effectiveness of table *C*. The performance of SubseqHash with fully random orders gives the highest probability (i.e. the Jaccard index) among possible orders, but the curves from ABC orders when *d *=* *11 and *d *=* *31 are very close to them, indicating that the ABC order (with large *d*) is nearly optimal.

### 3.3 Evaluating seeding methods

We discuss appropriate measures to evaluate seeding methods for tasks involving sequence comparison, such as sequence alignment, read mapping, and overlapping read detection. Despite variations in methods for these tasks, they all follow a two-step procedure, consisting of a “seeding step” and a “post-seeding step.” The seeding step treats the sequences/reads independently and typically applies a seeding method to sliding windows of a sequence/read, resulting in a list of seeds. Measures for this step include the running time of the seeding method, as well as the density of the seeds, defined as the number of seeds produced from a sequence divided by the length of the sequence.

The post-seeding step uses the generated seeds to compare sequences. We emphasize that in this step only the matched seeds between compared sequences are used, while unmatched seeds are discarded. For example, in the co-linear chaining approach for sequence alignment, the set of seed-matches serves as the input of the chaining algorithm. In the seed-and-extend scheme for mapping reads, each individual seed-match will be examined in the extension. When detecting overlapping reads, a pair of reads will be determined as “candidate” (which will be then subject to more fine-grained procedure such as chaining to decide overlapping) if there exists one (or more) seed-matches. Therefore, it is the quantity and quality of seed-matches, rather than that of seeds, that determine the running time of the post-seeding step and the accuracy of the outcomes.

In Sections 3.4 and 3.5, we report the density and running time of different seeding methods, as well as the quantity and quality of the resulting seed-matches to evaluate their impacts on the post-seeding step. In summary, SubseqHash runs much slower than substring-based methods, such as Minimizers. When repetitions are applied to SubseqHash (see Section 2.6), it generates a much larger number of seeds than Minimizers, requiring more memory to store the seeds. However, SubseqHash outperforms other methods in generating high-quality seed-matches, thereby improving the accuracy of the final outcomes. See below for detailed analysis.

### 3.4 Application: pairwise sequence alignment

We use simulations to test the performance of different methods with varying error rates. We simulate 10 pairs of sequences and report the average measures (described below). The first sequence in a pair is a random sequence of length *L *=* *100 000; the second sequence is obtained by applying an edit, with probability of *r* equally distributed to insertion, deletion, and substitution, independently on every position of the first sequence, where *r* is a parameter specifying the error rate. The ground-truth alignment is saved for evaluation (see below).

**Figure 3.**
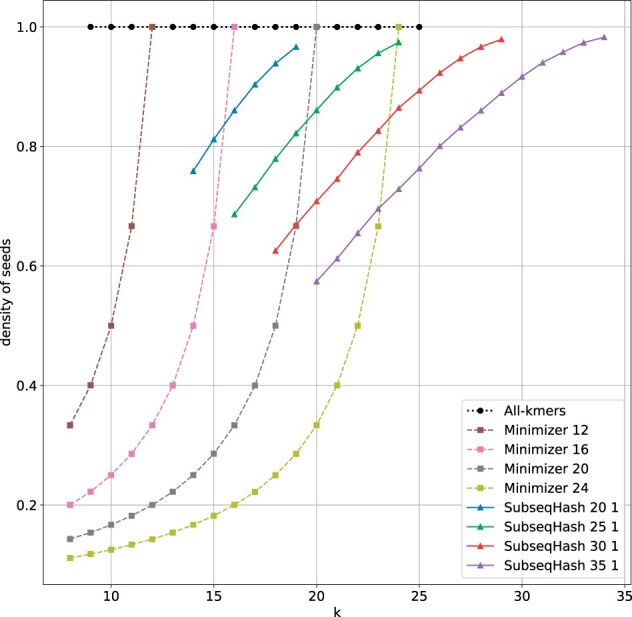
The density of different seeding methods on simulated sequences. In the legend, “all-kmers” means every single *k*mer is collected as seed, k=9,10,…,25. “Minimizer *n*” means a window size of *n*; with increasing density, the list of *k* used for each line is k=8,9,…,n−1. “SubseqHash *n t*” means a window size of *n* and repeating *t* times. For *n *=* *20, *k* is from 14 to 19; for *n *=* *25, *k* is from 16 to 24; for *n *=* *30, *k* is from 18 to 29; for *n *=* *35, *k* is from 20 to 34. For all methods, points with varying *k* but the same *n* are connected by lines. These parameters are also used in all experimental studies of Sections 3.4 and 3.5.

In Fig. 3, we present the density of SubseqHash and Minimizers using the simulated data described above. As expected, for any fixed window size *n*, the density of both methods decreases as the seed length *k* decreases, but the density of Minimizers decreases much more rapidly than that of SubseqHash. It should be noted that when repetitions are applied to SubseqHash, the density and total number of seeds should be multiplied by the number of repetitions. Furthermore, we provide a comparison of the running time of different seeding methods in Supplementary Tables S6–S9. Consistent with the theoretical analysis, SubseqHash typically runs 24–270 times slower than Minimizers.

**Figure 4.**
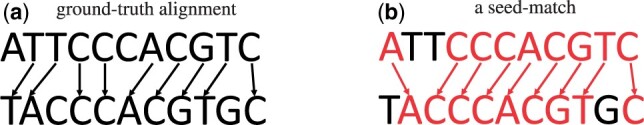
(a) The ground-truth alignment between two sequences; note that this alignment is not the most parsimonous (i.e. minimizing edit distance) alignment. (b) A seed-match where the identical seed is *ACCCACGTC*. Among the nine matched characters, five of them (55.6%) exist in the ground-truth alignment. Hence this seed-match is a true one.

We now assess the quality of resulting seed-matches. In either Minimizer or SubseqHash, a seed-match specifies an alignment among *k* identical characters. We define a seed-match to be “true” if at least 50% of the *k* aligned characters appear in the ground-truth; otherwise it is considered to be a “false” seed-match. See Fig. 4 for an example. We do not require all *k* aligned characters to agree with the ground-truth to be considered as a true seed-match as the ground-truth alignment may not reflect the most parsimonious alignment especially when the mutation rate is high. Nevertheless, 50% matched characters certainly indicate that the locations of two seeds are anchored correctly, which is adequate for downstream use.

**Figure 5.**
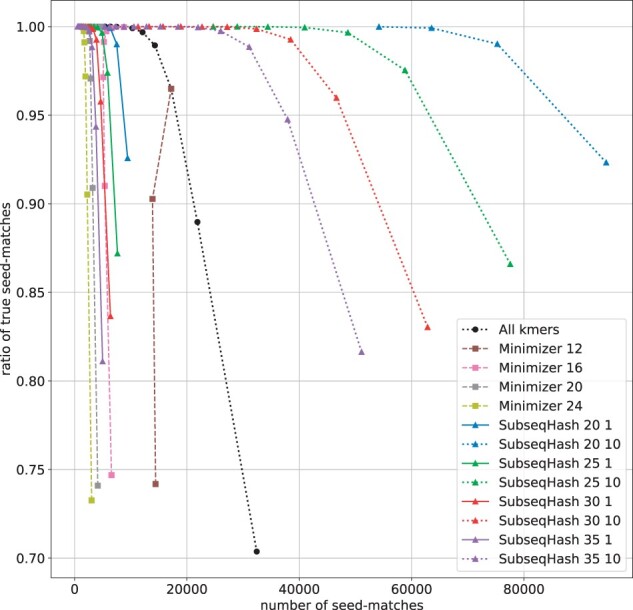
The number of seed-matches and the true seed-matches ratios on simulated sequences with error rate = 15%. Figure is cropped to only show the portion with high ratio (≥70%); complete results are shown in Supplementary Tables S2–S5. Results for error rates 5%, 10%, and 20% are available in Supplementary Fig. S7.

In Fig. 5 and Supplementary Fig. S7, we present the relationship between the number of seed-matches and the ratio of true seed-match ratio (defined as the number of true seed-matches divided by the number of seed-matches). As explained in Section 3.3, the number of seed-matches has a strong correlation with the execution time of the post-seeding step, while the true seed-match ratio reflects the accuracy of seed-matches. By comparing the *y*-coordinates of different methods at a fixed *x*-coordinate, one can assess their accuracy at the same level of running time. SubseqHash without repetitions demonstrates a similar performance to Minimizers in the range of small numbers of seed-matches. SubseqHash with repetitions achieves a much higher true ratio than all-*k*mers when the number of seed-matches falls between approximately 10 000 and 30 000. Beyond this range, SubseqHash and other methods are not comparable.

**Figure 6.**
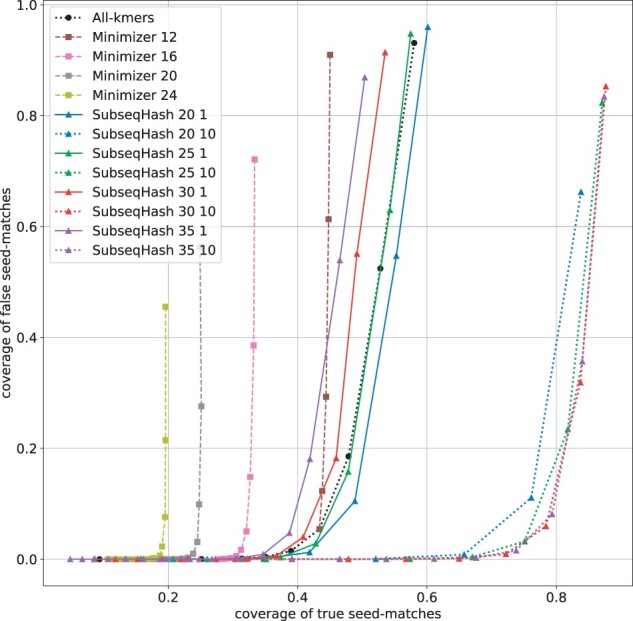
The coverages of true and false seed-matches for different seeding methods on simulated sequences with error rates =15%. Results for different error rates are available in Supplementary Fig. S8.

It is more desirable for a seeding method to generate true seed-matches that span a larger range of the sequence, rather than ones clustered together (Sahlin 2021). We say a character in a sequence is “covered” by a seed-match if it is one of its *k* aligned characters. The “coverage of true seed-matches” (true coverage for short) is the percentage of characters in both sequences that are covered by at least one true seed-match; the “coverage of false seed-matches” (false coverage for short) is defined in the same way but counting false seed-matches. Higher true coverage reflects higher sensitivity, and can facilitate downstream chaining procedure to produce more accurate and faster sequence alignment. Lower false coverage reduces the likelihood of producing incorrect alignments. We report the average true/false coverages of different methods in Fig. 6 and Supplementary Fig. S8. A single run of SubseqHash outperforms (i.e. higher true coverage at the same false coverage) Minimizer and all-*k*mers when error rate is 5% or 10%, and achieves similar performance at 15% and 20% error rates. SubseqHash with 10 repetitions outperforms others by a large margin at all error rates: specifically, the highest true coverage achieved by Minimizer/all-*k*mers at a false coverage lower than 5% are 85.1%, 61.4%, 38.9%, and 23.1%, respectively, for the 4 error rates, while the numbers for SubseqHash are 98.0%, 90.8%, 75.3%, and 55.0%, respectively.

### 3.5 Application: read mapping

We then compare SubseqHash with other seeding methods on mapping reads to the reference genome. We utilize three real PacBio datasets from Berlin et al. (2015) on *Escherichia coli* (SRX533603), *Saccharomyces cerevisiae* (SRX533604), and *Drosophila melanogaster* (SRX499318). To construct a ground-truth for evaluation, we align the reads to the corresponding reference genomes using minimap2 (Li 2018) with the default parameters (-cx map-pb). For reads that have a mapped region of at least 2000 bp and a mapping quality of at least 10, we trim the read to only keep the mapped portion. Reads with multiple qualified mappings are discarded. This produces the input reads and their ground-truth alignments. To eliminate the potential biases in the ground-truth created by minimap2, which internally uses Minimizers for seeding, we include a simulated dataset obtained with PBSIM2 (Ono et al. 2021) using the same statistics as the PacBio *D.melanogaster* dataset. A total of 287 648 reads are simulated from the X chromosome, with ground-truth alignment saved from simulation. We randomly sample 1000 reads from each of the four datasets for this experiment. The same set of seeding methods (Minimizer, all-*k*mers, and SubseqHash) is applied to generate seeds for both reads and the reference genomes. The density of different methods is illustrated in Supplementary Figs S9 and S10, which show very similar results with Fig. 3.

**Figure 7.**
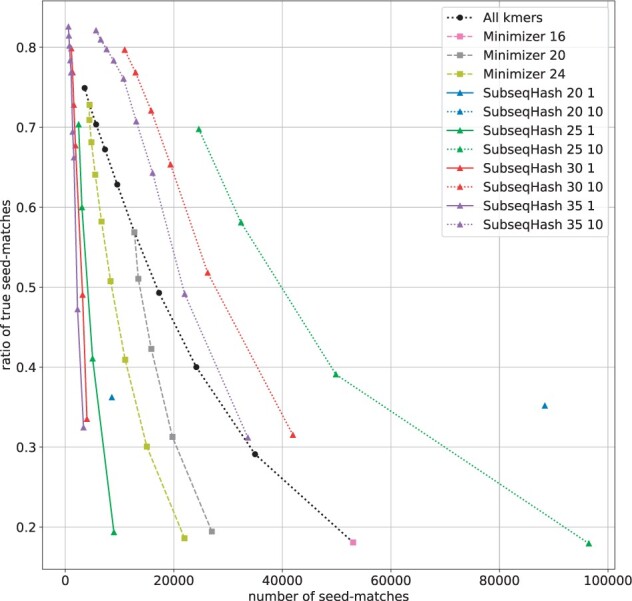
The number of seed-matches and the ratio of true seed-matches for different seeding methods evaluated on *D.melanogaster* SRX499318 dataset. Figure is cropped to show high ratios (≥15%); complete results are given in Supplementary Tables S10–S13. Results for other datasets are shown in Supplementary Fig. S11.

**Figure 8.**
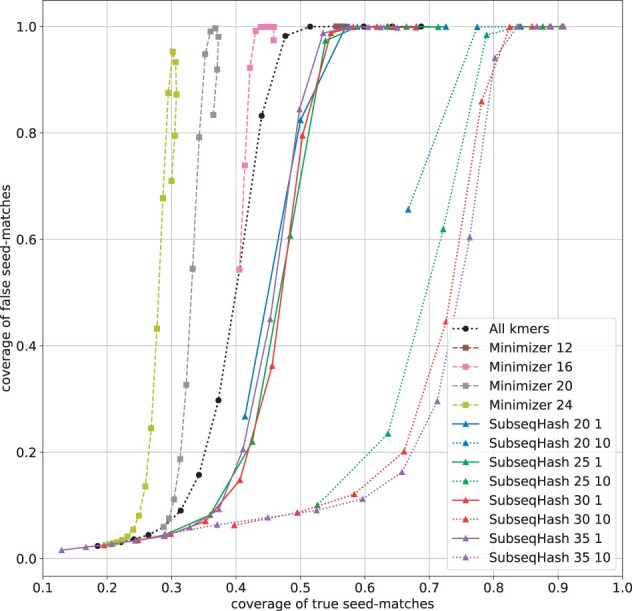
The coverages of true and false seed-matches for different seeding methods on *D.melanogaster* SRX499318 dataset. Results for other datasets are available in Supplementary Fig. S12.

To assess the quality of seed-matches, the same definitions of true and false seed-match used for pairwise sequence alignment (Section 3.4) is also used in this experiment. When calculating the true/false coverages, only the covered characters on reads are considered (instead of on both the reads and the reference genomes). Figure 7 and Supplementary Fig. S11 show the number of seed-matches and the ratio of true seed-matches for different methods averaged over the 1000 reads. At the same level of seed-matches, SubseqHash with 10 repetitions can achieve much higher ratio of true seed-matches than all-*k*mers and Minimizers, verifying the effectiveness of repetitions for sequence mapping. Figure 8 and Supplementary Fig. S12 compare the true/false coverages of different methods. SubseqHash (without repeating) can obtain higher true coverage on the same foot of false coverage than all-*k*mers and Minimizers. Again SubseqHash with repeating 10 times outperforms all others substantially. To give some concrete numbers, the highest true coverage achieved by Minimizer/all-*k*mers at a false coverage lower than 10% are 38.2%, 39.0%, 31.4%, and 34.2%, respectively, for the four datasets, while the numbers for SubseqHash are 74.5%, 67.3%, 52.5%, and 57.5%, respectively.

### 3.6 Application: overlap detection

State-of-the-art methods for genome assembly using long-reads sequencing data often rely on an accurate overlap graph, in which vertices are reads and overlapping reads are connected with edges. A straightforward approach for constructing the overlap graph given a set of sequences (i.e. long reads) is performing all-versus-all comparisons, but it certainly does not scale. Seeding methods can be used to detect overlapping pairs while being able to scale. More specifically, a seeding method first transforms each sequence into seeds, then reports pairs of sequences that have at least one seed-match as candidate overlapping pairs. This is certainly a coarse model as for a real overlap detection tool, one would usually perform multiple steps of seed-preprocessing, such as subsampling and filtering; then in the detection phase (post-seeding step), different thresholds for the number of seed-matches and location information of the seeds can be used; lastly, the candidate overlapping pairs are often verified with a fine-grained comparison (e.g. a local alignment) before the final output. All these steps make the overlap results more accurate, but because they can be applied regardless of the seeding methods used, we opt to omit them in this experiment so we can focus on a direct comparison of different seeding methods.

We use the same four datasets in Section 3.5 in this experiment. We sample 10 000 reads from each dataset; a pair of reads are considered truly overlapping (i.e. ground-truth) if their mapped regions on the reference genome overlap by at least 15 bp. To measure the candidate overlapping pairs reported by a seeding method, we define sensitivity as the fraction of ground-truth pairs that are identified by a seeding method; define precision as the fraction of all reported pairs that are correct according to the ground-truth.

**Figure 9.**
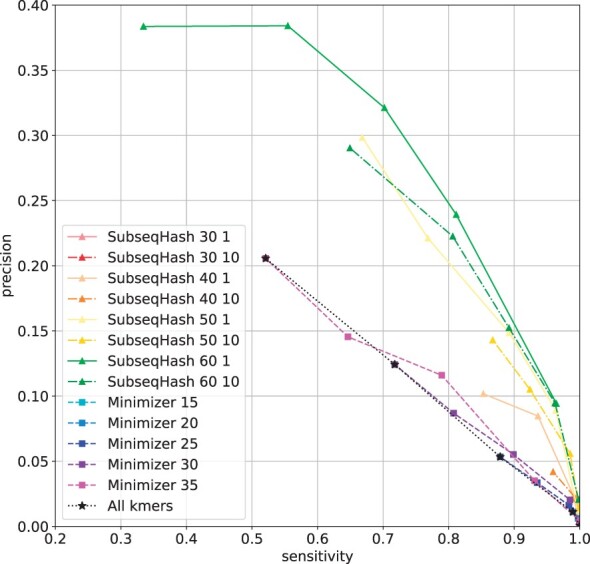
Overlap detection results on reads sampled from *D.melanogaster* SRX499318 dataset. Results for other datasets are available in Supplementary Fig. S13.

The precision-sensitivity curves for different methods are shown in Fig. 9 and Supplementary Fig. S13. Comparing with seeding for sequence alignment and read mapping, here we use a larger window size mainly to reduce false pairs. For each window size *n* used in Minimizers, six seed lengths *k* evenly spaced between 10 and *n* are included (when *k *=* n*, all-*k*mers are picked as seeds). For each *n* used in SubseqHash, the seed lengths k=⌊0.65n⌋, ⌊0.7n⌋, ⌊0.75n⌋, ⌊0.8n⌋, ⌊0.85n⌋ are tested. We include the results of SubseqHash without repetition and of 10 repeats for each of the parameters above. When repetitions apply, the overlapping pairs are simply the union of all 10 runs. Observe that on all four datasets, SubseqHash without repetition already shows better accuracy (i.e. higher precision at the same sensitivity level) than Minimizers and all-*k*mers with a few exceptions at sensitivity near 1.0. In this region, both methods suffer from extremely low precision, which indicates that refining steps are necessary and raw seeds comparison at sensitivity close to 1.0 may not be truly informative. With 10 repetitions, the sensitivity of SubseqHash is significantly boosted while outperforming Minimizers and all-*k*mers substantially. For example, when the sensitivity is set to be at least 80%, the highest precisions achieved by Minimizer/all-*k*mers for the four datasets are 62.9%, 38.9%, 8.7%, and 11.7%, while the numbers for SubseqHash are 85.9%, 78.0%, 23.9%, and 41.7%, respectively.

## 4 Discussion

We investigated SubseqHash, a new approach that uses the minimized subsequence as seed. We figured that the probability of hash collision is determined by the shared order. We therefore studied this core algorithmic formulation: seek an order *π* over all strings of length *k* such that *π* is “as random as possible” and that an efficient algorithm that finds the smallest subsequence (according to *π*) in a string (of length *n*) can be designed. We gave a practical solution for this formulation, consisting of the so-called ABC order together with a dynamic programming algorithm runs in *O*(*nkd*) time to find the minimized subsequence under an ABC order (where d≥1 can be picked by users). We demonstrated that nearby strings in an ABC order are distant from each other, a property exhibited in a fully random order, and that the probability of hash collision with an ABC order is close to the Jaccard index, achievable when a fully random order is used.

The superiority of SubseqHash over substring-based methods is in 3-folds. First, SubseqHash tolerates errors while substring-based methods require exact matches. Second, the probability of hash collision of SubseqHash is higher than that of Minimizer (when extracting seeds of the same length). Third, the performance of SubseqHash can be substantially boosted through repetition while for Minimizer this is not as practical. These merits make SubseqHash a more suitable choice for seeding sequencing data with high error/mutation rates.

We showed that SubseqHash coupled with the ABC order substantially outperformed Minimizer in generating high-quality seed-matches for three applications, sequence alignment, read mapping, and overlap detection. We emphasize that these experiments were designed for a direct comparison between different seeding methods, and therefore the evaluations were conducted at the level of seeds, rather than evaluating the eventual outcomes (e.g. alignments or the overlap graph). As seeding is a key step involved in these applications, we expect SubseqHash will be widely adapted and incorporated to improve their accuracies on the analysis of third-generation sequencing data.

Our algorithm to find a single seed takes *O*(*nkd*) time, which is much slower than Minimizer that takes amortized *O*(1) time to find a seed in a window. We note that the seeding step usually takes less time than the post-seeding step especially in applications that require all-pairs comparisons, as seeding scales linearly with respect to the number of sequences. Users may choose a smaller *d*, say *d *=* *4 or even *d *=* *1, to gain a speedup at the cost of slightly decrease of sensitivity. Interesting future directions include accelerating the current algorithm using techniques, such as A∗ heuristic searching (Ivanov et al. 2022) and parallel algorithms that have been successfully used to speed up dynamic programming algorithms. We also believe the core algorithmic formulation (stated in the first paragraph of this section), which we find fascinating, can be further improved in achieving faster algorithm and/or higher probability of hash collision.

## Supplementary Material

btad218_Supplementary_Data
