# Supplementary material for: Seeding with minimized subsequence
Source: Bioinformatics. 2023 Jun 30;39(Suppl 1):i232–41. doi: 10.1093/bioinformatics/btad218 (PMC10311335; doi:10.1093/bioinformatics/btad218)
Supplement: btad218_Supplementary_Data [file btad218_supplementary_data.pdf]

Supplementary Materials for
"Seeding with Minimized Subsequence"

Xiang Li[1], Qian Shi[1,†], Ke Chen[1,†], and Mingfu Shao[1,2,*]

[1]Department of Computer Science and Engineering, School of Electronic Engineering and Computer Science, The Pennsylvania State University
[2]Huck Institutes of the Life Sciences, The Pennsylvania State University

March 24, 2023

List of Supplementary Notes

1 Jaccard for Subsequences and Substrings 3
2 Expectation of Distinct Subsequences 6
3 An Example for the ABC Order 8

List of Supplementary Figures

1 Estimated probability of hash collision for $n = 20$ 4
2 Estimated probability of hash collision for $n = 30$ 5
3 The AMNED of different orders 10
4 Probability of hash collision for $n = 20$ 11
5 Probability of hash collision for $n = 30$ 12
6 Density of seeds in pairwise sequence alignment 13
7 Seed-matches in pairwise sequence alignment 14
8 True/false coverage in pairwise sequence alignment 15
9 Density of seeds generated from reference genome 16
10 Density of seeds generated from reads 17
11 Seed-matches in read mapping 18
12 True/false coverage in read mapping 19
13 Overlap detection results on reads sampled from 3 datasets. 20

List of Supplementary Tables

1 An example of random tables used in an ABC order 9
2 SubseqHash seed-matches in pairwise sequence alignment 21
3 SubseqHash with repetition seed-matches in pairwise sequence alignment 22
4 Kmer seed-matches in pairwise sequence alignment 23
5 Minimizer seed-matches in pairwise sequence alignment 24

[†]These authors contributed equally to this work.
[*]Correspondence should be addressed to mxs2589@psu.edu.

6 Running time of subseqhash seeding in pairwise sequence alignment 25
7 Running time of subseqhash seeding with repeating in pairwise sequence alignment . 26
8 Running time of kmer seeding in pairwise sequence alignment 27
9 Running time of minimizer seeding in pairwise sequence alignment 28
10 SubseqHash seed-matches in read mapping 29
11 Subseqhash with repetition seed-matches in read mapping 30
12 Kmer seed-matches in read mapping 31
13 Minimizer seed-matches in read mapping 32
14 Running time of subseqhash seeding in read mapping 33
15 Running time of subseqhash seeding with repeating in read mapping 34
16 Running time of kmer seeding in read mapping 35
17 Running time of minimizer seeding in read mapping 36

Supplementary Note 1: Jaccard for Subsequences and Substrings

Recall that we use $S_k(x)$ to denote the set of subsequences of length k of string $x \in \Sigma^n$. We further use $S'_k(x)$ to denote the set of substrings of length k of x. Given two strings x and y, the Jaccard index for their subsequences and substrings are defined as $J(x,y) := |S_k(x) \cap S_k(y)|/|S_k(x) \cup S_k(y)|$ and $J'(x,y) := |S'_k(x) \cap S'_k(y)|/|S'_k(x) \cup S'_k(y)|$, respectively. We denote by $dist(x,y)$ the edit distance of two strings x and y. We aim to estimate the expected Jaccard index for two random strings of length n with a fixed edit distance d, namely, $\mathbb{E}_{x,y|dist(x,y)=d}J(x,y)$ and $\mathbb{E}_{x,y|dist(x,y)=d}J'(x,y)$.

We use $E_k^n := \mathbb{E}_{x\in\Sigma^n}|S_k(x)|$ to denote the expected number of distinct subsequences of length k over all strings of length n. A recursive formula for E_k^n is derived in the Appendix B. To calculate $|S_k(x) \cap S_k(y)|$, consider the optimal alignment of x and y. Given that $dist(x,y) = d$, the length of the longest common subsequence (LCS) between x and y is at least $n - d$. Therefore, the expectation of $|S_k(x) \cap S_k(y)|$ given that $dist(x,y) = d$, i.e., $\mathbb{E}_{x,y|dist(x,y)=d}|S_k(x) \cap S_k(y)|$, can be estimated by E_k^{n-d}. For the denominator, we estimate the expectation of $|S_k(x) \cup S_k(y)| = |S_k(x)| + |S_k(y)| - |S_k(x) \cap S_k(y)|$ by $2E_k^n - E_k^{n-d}$. Combined, we have

$$\mathbb{E}_{x,y|dist(x,y)=d}J(x,y) \approx \frac{E_k^{n-d}}{2E_k^n - E_k^{n-d}}.$$

To estimate $\mathbb{E}_{x,y|dist(x,y)=d}J'(x,y)$, we assume all substrings of length k in x (resp. in y), are distinct, which gives $|S'_k(x)| = |S'_k(y)| = n - k + 1$. For simplicity, we treat the d edits between x and y as being caused by d mutations occurred on d uniformly randomly chosen positions on x. Then $\mathbb{E}_{x,y|dist(x,y)=d}|S'_k(x) \cap S'_k(y)|$ can be estimated by the expected number of intact substrings of length k in x through the mutations. Because the probability that one substring remains unchanged is $\binom{n-k}{d}/\binom{n}{d}$, the expected number of unchanged substrings is $(n-k+1)\binom{n-k}{d}/\binom{n}{d}$. Therefore,

$$\mathbb{E}_{x,y|dist(x,y)=d}J'(x,y) \approx \frac{\binom{n-k}{d}}{2\binom{n}{d} - \binom{n-k}{d}}.$$

We calculate the estimated Jaccard index for different edit distances and show them in Figure 1 and Figure 2. It is clear that the Jaccard index for subsequences is larger than that for substrings. This reflects the major advantage of SubseqHash, which also motivates its design, that it is more sensitive in comparing sequences with relatively high error/mutation rate.

Note that above theoretical estimations for Jaccard index are made under several assumptions in order to derive them. For a more realistic comparison we also estimate them using simulation data (see Section 3.2 for more details).

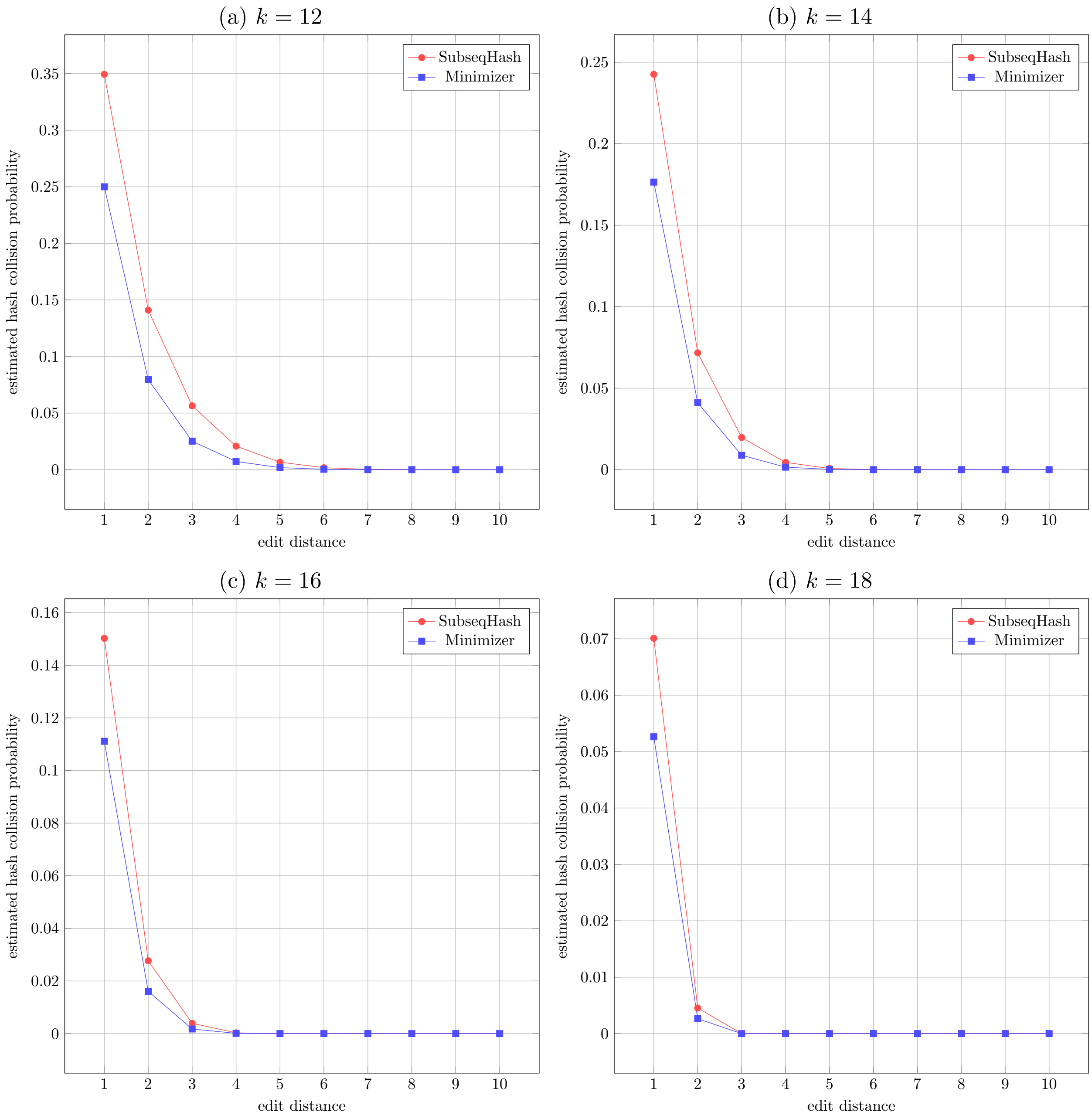

Supplementary Figure 1: For $n = 20$, SubseqHash has higher estimated hash collision probabilities than Minimizer.

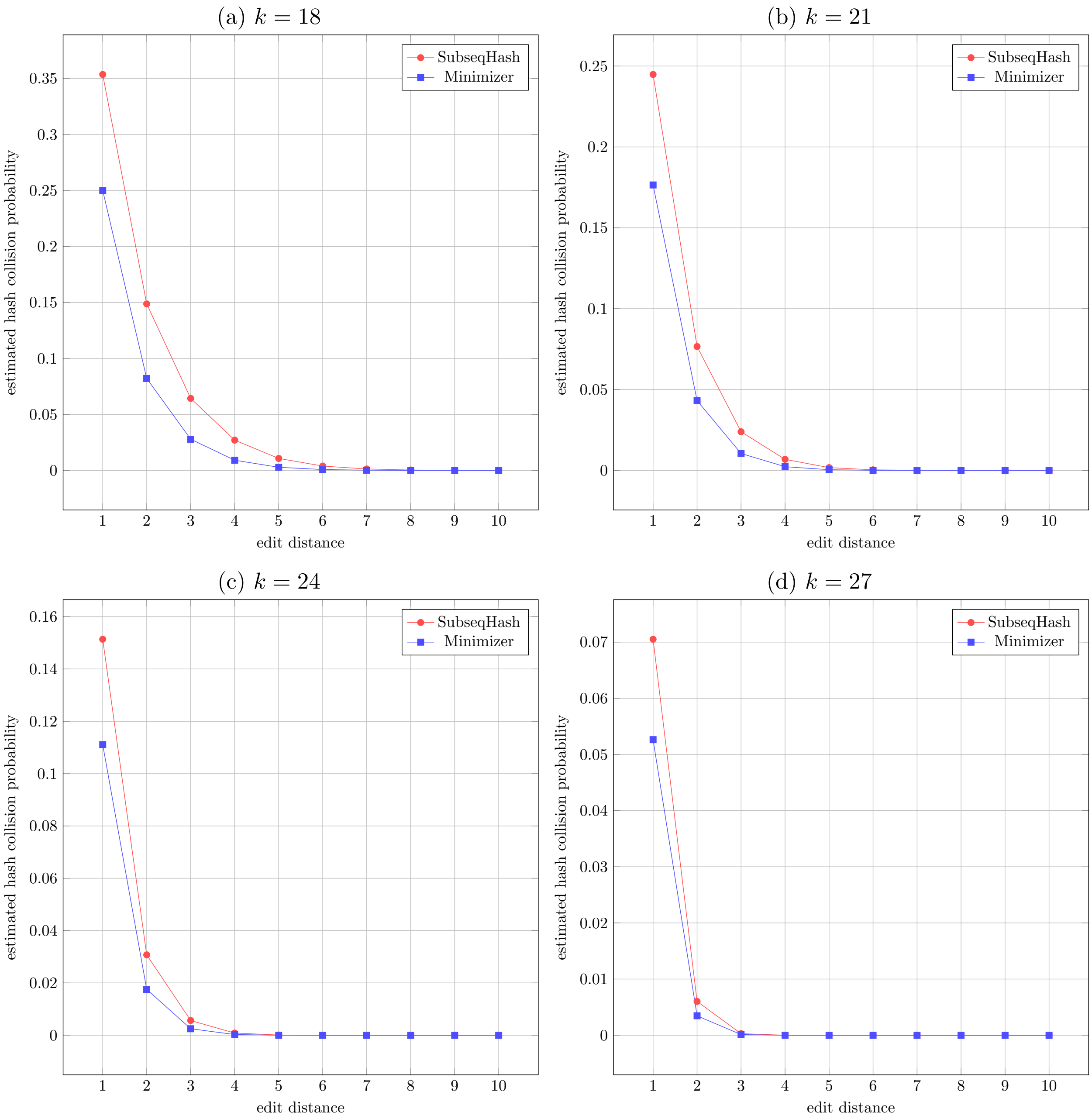

Supplementary Figure 2: For $n = 30$, SubseqHash has higher estimated hash collision probabilities than Minimizer.

Supplementary Note 2: Expectation of Distinct Subsequences

We extend [1] to calculate average number of distinct subsequences in a uniformly distributed random string over alphabet Σ. We call a subsequence of length k as a k-subsequence, for the sake of simplicity. For a string $s \in \Sigma^n$, we define $\sigma_k(s) := |S_k(s)|$, i.e., the number of distinct k-subsequence of s. Given a string $s = (s_1 s_2 \dots s_n)$, we denote substring $(s_i s_{i+1} \dots s_j)$ by $s[i, j]$.

For character $c \in \Sigma$, let d_c be the number of characters before the first appearance of c in s. If $c \notin s$, $d_c = n$ and then $\sigma_k(s[d_c + 1, n]) = 0$. To derive a recurrence, we consider distinct k-subsequence starting at a specific character c. Then $\sigma_k(s)$ is equal to the sum of the number of distinct k-subsequence starting from all possible $c \in \Sigma$, formally,

$$\sigma_b(k) = \sum_{c\in\Sigma} \sigma_{k-1}(s[d_c + 1, n]).$$

Let $a = |\Sigma|$. For each $c \in \Sigma$, when $d_c < n$, it is with probability of $(1 - 1/a)^{d_c}/a$ that the first appearance of c is at position $d_c + 1$. Also, the probability that $c \notin s$ is $(1 - 1/a)^n$. Recall that we use $\mathbb{E}_k^n$ to denote the expectation of $\sigma_k(s)$ over all strings $s \in \Sigma^k$. Since every $c \in \Sigma$ has the same probability to appear at any position of s, we have:

$$\begin{aligned} E_k^n &= \mathbb{E}_{s\in\Sigma^n} \sigma_k(s) \\ &= \left(\sum_{d_c \geq 0}^{n-1} \left(1 - \frac{1}{a}\right)^{d_c} \cdot \frac{1}{a} \cdot E_{k-1}^{n-d_c-1} \right) \cdot a \\ &= \sum_{d_c \geq 0}^{n-1} \left(1 - \frac{1}{a}\right)^{d_c} \cdot E_{k-1}^{n-d_c-1} \end{aligned} \tag{1}$$

Given that $\mathbb{E}_k^n = 0$ for $k > n$, we further have:

$$E_k^n = \sum_{d_c \geq 0}^{\infty} \left(1 - \frac{1}{a}\right)^{d_c} \cdot E_{k-1}^{n-d_c-1} \tag{2}$$

Notice that:

$$\begin{aligned} \left(1 - \frac{1}{a}\right) E_k^{n-1} &= \sum_{d_c \geq 0}^{\infty} \left(1 - \frac{1}{a}\right)^{d_c} \cdot E_{k-1}^{n-d_c-2} \cdot \left(1 - \frac{1}{a}\right) \\ &= \sum_{d_c \geq 0}^{\infty} \left(1 - \frac{1}{a}\right)^{d_c+1} \cdot E_{k-1}^{n-(d_c+1)-1} \\ &= \sum_{d_c \geq 1}^{\infty} \left(1 - \frac{1}{a}\right)^{d_c} \cdot E_{k-1}^{n-d_c-1} \\ &= \sum_{d_c \geq 0}^{\infty} \left(1 - \frac{1}{a}\right)^{d_c} \cdot E_{k-1}^{n-d_c-1} - E_{k-1}^{n-1} \\ &= E_k^n - E_{k-1}^{n-1}. \end{aligned}$$

This gives

$$E_k^n = E_{k-1}^{n-1} + \left(1 - \frac{1}{a}\right) E_k^{n-1} \tag{3}$$

References

[1] Michael J Collins. The number of distinct subsequences of a random binary string. *arXiv*, page 1310.7288, 2013.

Supplementary Note 3: An Example for the ABC Order

We give an example for the ABC order, determined by the three random tables shown in Supplementary Table 1 with $k = 6$, $d = 5$, and $\Sigma = \{A, C, G, T\}$. The score of $\boldsymbol{z}$ = CTAACT can be calculated according to the tables following the recurrences, with detailed given below.

$$
\begin{aligned}
\psi_0 &= 0,\\
\psi_1 &= (\psi_0 + C[1][\text{C}]) \bmod 5 = (0+3) \bmod 5 = 3,\\
\psi_2 &= (\psi_1 + C[2][\text{T}]) \bmod 5 = (3+4) \bmod 5 = 2,\\
\psi_3 &= (\psi_2 + C[3][\text{A}]) \bmod 5 = (2+1) \bmod 5 = 3,\\
\psi_4 &= (\psi_3 + C[4][\text{A}]) \bmod 5 = (3+0) \bmod 5 = 3,\\
\psi_5 &= (\psi_4 + C[5][\text{C}]) \bmod 5 = (3+0) \bmod 5 = 3,\\
\psi_6 &= (\psi_5 + C[6][\text{T}]) \bmod 5 = (3+2) \bmod 5 = 0.
\end{aligned}
$$

$$
\begin{aligned}
\omega_0 &= 0,\\
\omega_1 &= \omega_0 \cdot B[1][\psi_1][\text{C}]_1 + A[1][\psi_1][\text{C}] \cdot B[1][\psi_1][\text{C}]_2\\
&= 0 \cdot (+1) + 97.70 \cdot (+1) = 97.70,\\
\omega_2 &= \omega_1 \cdot B[2][\psi_2][\text{T}]_1 + A[2][\psi_2][\text{T}] \cdot B[2][\psi_2][\text{T}]_2\\
&= 97.70 \cdot (-1) + 95.09 \cdot (-1) = -192.79,\\
\omega_3 &= \omega_2 \cdot B[3][\psi_3][\text{A}]_1 + A[3][\psi_3][\text{A}] \cdot B[3][\psi_3][\text{A}]_2\\
&= -192.79 \cdot (-1) + 47.63 \cdot (+1) = 240.42,\\
\omega_4 &= \omega_3 \cdot B[4][\psi_4][\text{A}]_1 + A[4][\psi_4][\text{A}] \cdot B[4][\psi_4][\text{A}]_2\\
&= 240.42 \cdot (+1) + 97.16 \cdot (+1) = 337.58,\\
\omega_5 &= \omega_4 \cdot B[5][\psi_5][\text{C}]_1 + A[5][\psi_5][\text{C}] \cdot B[5][\psi_5][\text{C}]_2\\
&= 337.58 \cdot (+1) + 76.62 \cdot (+1) = 414.20,\\
\omega_6 &= \omega_5 \cdot B[6][\psi_6][\text{T}]_1 + A[6][\psi_6][\text{T}] \cdot B[6][\psi_6][\text{T}]_2\\
&= 414.20 \cdot (+1) + 59.33 \cdot (+1) = 473.53.
\end{aligned}
$$

So $\pi(\text{CTAACT}) = (0, 473.53)$. Similarly, we get $\pi(\text{CCAACT}) = (2, -98.78)$ and $\pi(\text{CCAACA}) = (0, -64.07)$.

Supplementary Table 1: An example of random tables used in an ABC order. Entries in table A are drawn from $[10, 100]$ instead of $\left[2^{30}, 2^{31}\right]$ in the implementation.

(a) Table A

		σ			
i	j	A	C	G	T
1	0	15.03	47.83	21.42	89.56
	1	92.79	80.235	12.00	54.64
	2	53.08	29.73	12.55	30.56
	3	80.63	97.70	64.14	67.43
	4	40.73	41.58	45.97	39.75
2	0	78.72	48.57	72.67	57.26
	1	87.31	95.57	28.07	34.98
	2	45.07	89.50	45.59	95.09
	3	24.02	49.49	42.99	53.58
	4	19.42	60.237	30.71	80.46
3	0	16.84	60.57	50.78	53.82
	1	81.80	40.51	93.28	71.85
	2	52.69	31.75	15.14	89.07
	3	47.63	33.81	27.73	21.42
	4	68.86	32.58	28.58	14.42
4	0	77.01	95.37	96.85	42.28
	1	90.49	75.14	67.23	56.71
	2	36.77	10.68	52.62	10.14
	3	97.16	90.68	29.78	85.49
	4	24.95	93.75	11.35	14.78
5	0	83.04	95.93	54.94	48.79
	1	63.22	67.58	65.74	83.46
	2	80.88	62.41	70.70	41.59
	3	66.80	76.62	67.62	34.46
	4	90.49	35.97	18.55	32.27
6	0	28.82	66.07	21.40	59.33
	1	97.79	23.66	26.31	68.52
	2	59.50	95.04	19.75	63.53
	3	37.16	32.74	21.77	90.85
	4	73.59	45.42	54.51	36.65

(b) Table B

		σ			
i	j	A	C	G	T
1	0	$(+1,+1)$	$(-1,-1)$	$(-1,+1)$	$(+1,-1)$
	1	$(-1,-1)$	$(+1,+1)$	$(-1,+1)$	$(+1,-1)$
	2	$(+1,+1)$	$(-1,-1)$	$(-1,+1)$	$(+1,-1)$
	3	$(-1,+1)$	$(+1,+1)$	$(-1,-1)$	$(+1,-1)$
	4	$(-1,-1)$	$(-1,+1)$	$(+1,+1)$	$(+1,-1)$
2	0	$(+1,+1)$	$(-1,-1)$	$(+1,-1)$	$(-1,+1)$
	1	$(-1,+1)$	$(+1,-1)$	$(+1,+1)$	$(-1,-1)$
	2	$(+1,+1)$	$(-1,+1)$	$(+1,-1)$	$(-1,-1)$
	3	$(+1,-1)$	$(+1,+1)$	$(-1,+1)$	$(-1,-1)$
	4	$(-1,+1)$	$(+1,-1)$	$(-1,-1)$	$(+1,+1)$
3	0	$(-1,-1)$	$(-1,+1)$	$(+1,-1)$	$(+1,+1)$
	1	$(+1,-1)$	$(-1,-1)$	$(+1,+1)$	$(-1,+1)$
	2	$(-1,-1)$	$(-1,+1)$	$(+1,+1)$	$(+1,-1)$
	3	$(-1,+1)$	$(-1,-1)$	$(+1,+1)$	$(+1,-1)$
	4	$(+1,-1)$	$(+1,+1)$	$(-1,+1)$	$(-1,-1)$
4	0	$(-1,+1)$	$(-1,-1)$	$(+1,+1)$	$(+1,-1)$
	1	$(+1,-1)$	$(-1,+1)$	$(-1,-1)$	$(+1,+1)$
	2	$(-1,+1)$	$(+1,-1)$	$(+1,+1)$	$(-1,-1)$
	3	$(+1,+1)$	$(-1,-1)$	$(+1,-1)$	$(-1,+1)$
	4	$(-1,-1)$	$(+1,+1)$	$(+1,-1)$	$(-1,+1)$
5	0	$(+1,+1)$	$(-1,+1)$	$(+1,-1)$	$(-1,-1)$
	1	$(+1,-1)$	$(-1,-1)$	$(-1,+1)$	$(+1,+1)$
	2	$(+1,+1)$	$(-1,+1)$	$(+1,-1)$	$(-1,-1)$
	3	$(-1,-1)$	$(+1,+1)$	$(-1,+1)$	$(+1,-1)$
	4	$(+1,+1)$	$(-1,+1)$	$(+1,-1)$	$(-1,-1)$
6	0	$(+1,-1)$	$(-1,-1)$	$(-1,+1)$	$(+1,+1)$
	1	$(-1,+1)$	$(-1,-1)$	$(+1,+1)$	$(+1,-1)$
	2	$(-1,+1)$	$(+1,+1)$	$(-1,-1)$	$(+1,-1)$
	3	$(-1,+1)$	$(+1,-1)$	$(-1,-1)$	$(+1,+1)$
	4	$(-1,+1)$	$(+1,+1)$	$(-1,-1)$	$(+1,-1)$

(c) Table C

	σ			
i	A	C	G	T
1	2	3	1	4
2	3	1	2	4
3	1	0	4	3
4	0	1	4	3
5	3	0	4	2
6	0	3	4	2

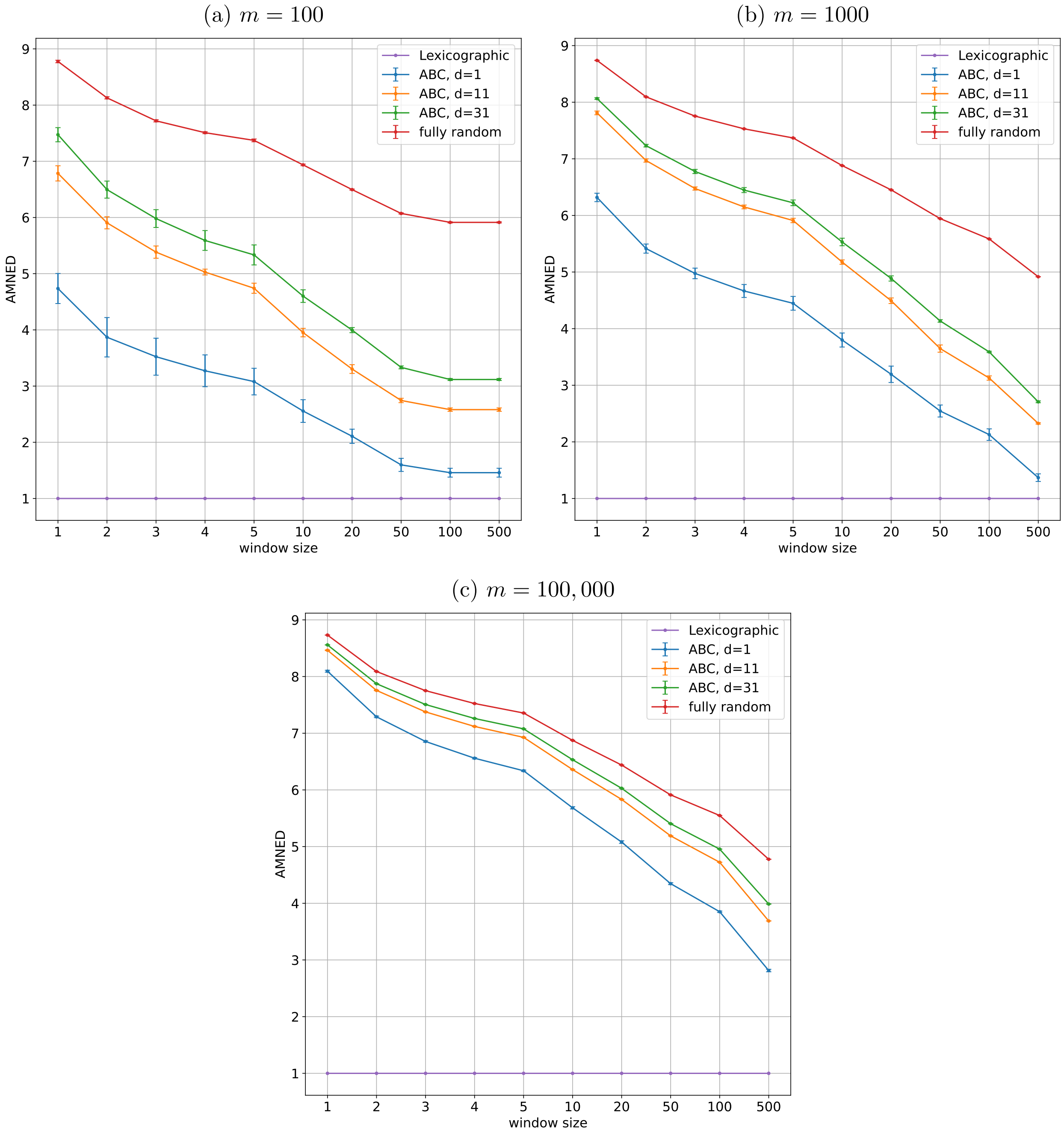

Supplementary Figure 3: The AMNED of different orders over strings of length $k = 15$ evaluated with varying w (x-axis) and m (3 subfigures with m being 100, 1000, and $100,000$). The point and error bar show the mean and standard deviation over 10 individual runs.

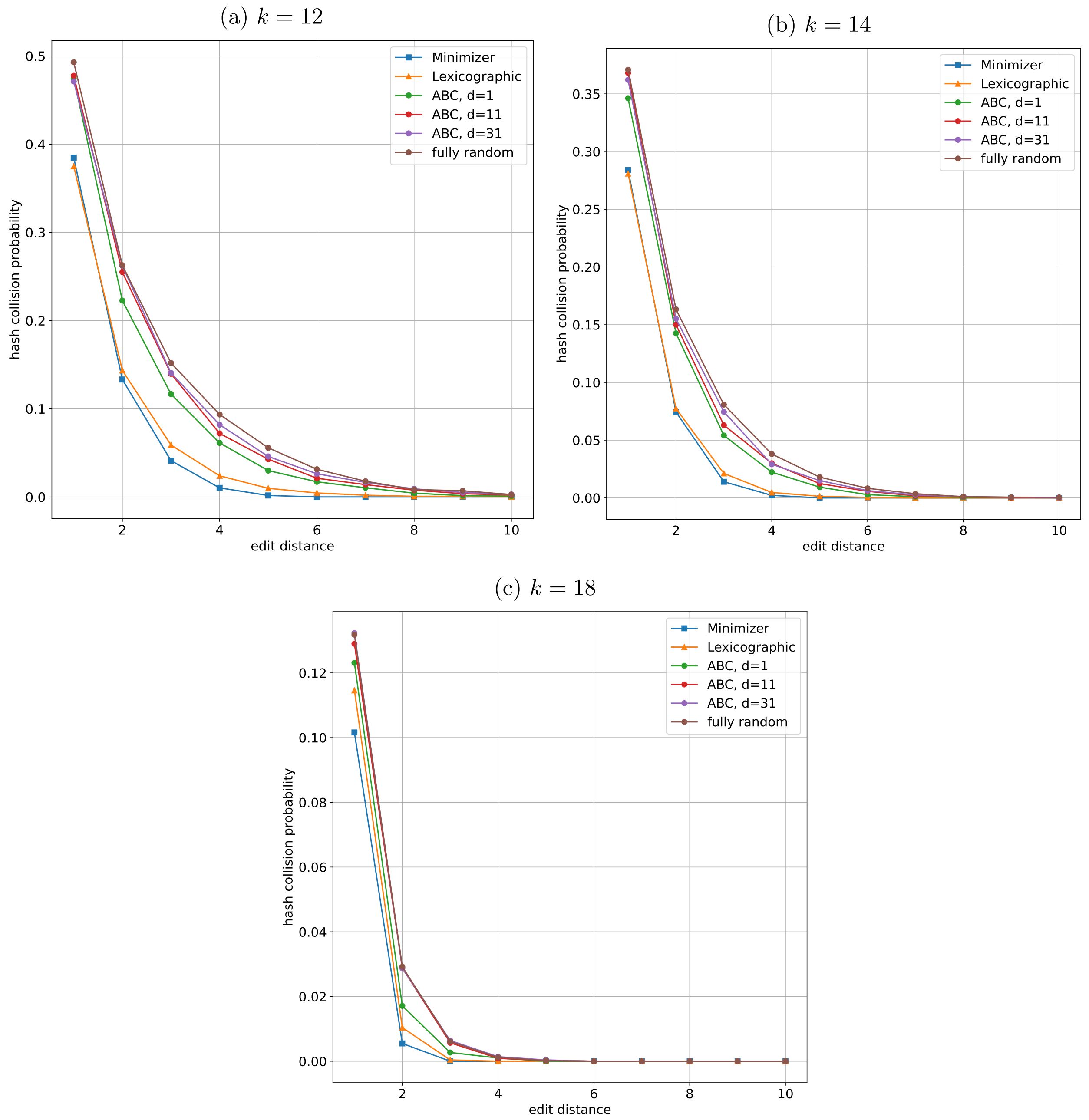

Supplementary Figure 4: The probability of hash collision estimated, using simulations, for different seeding methods with $n = 20$ and $k = 12, 14, 18$.

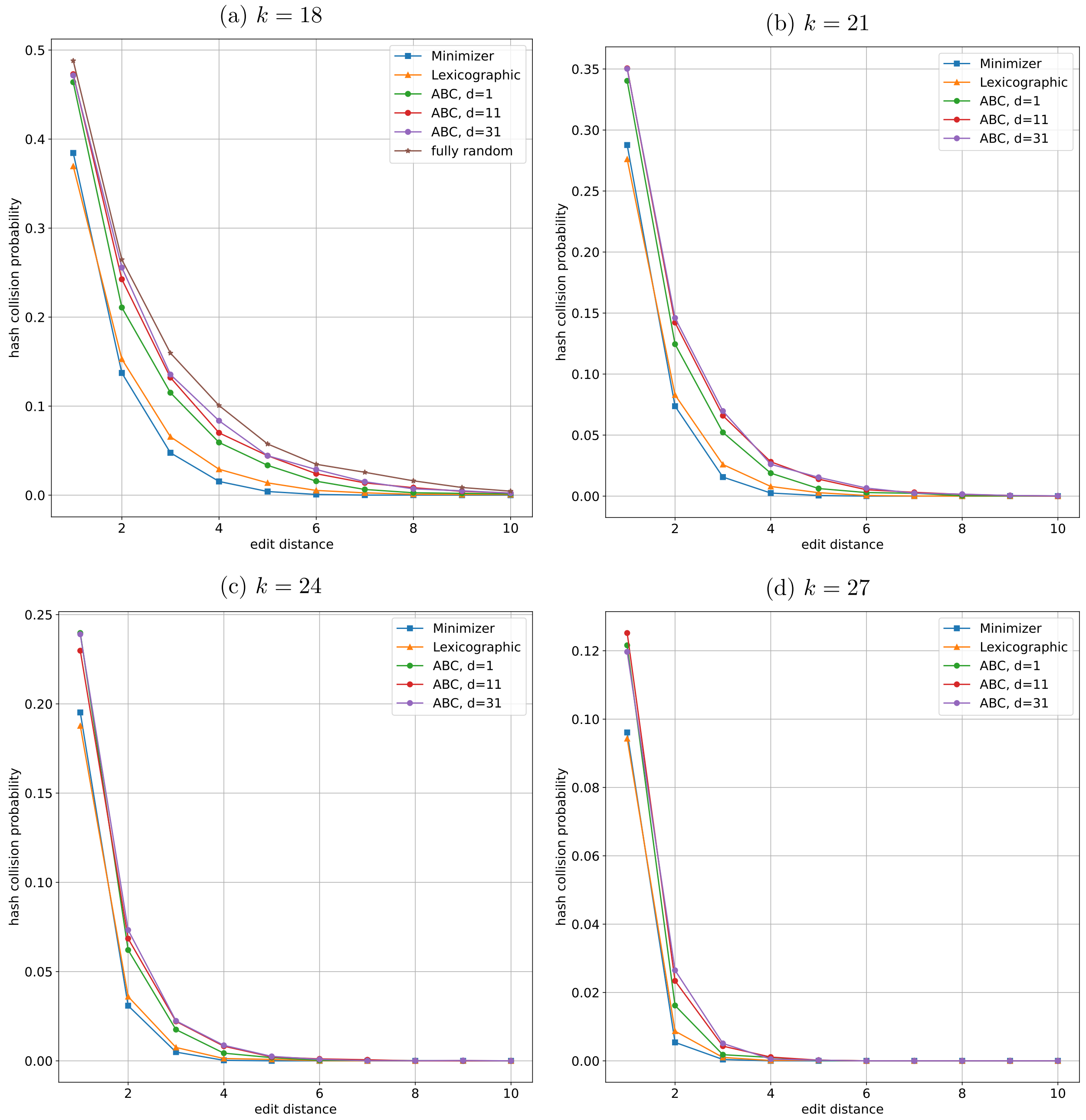

Supplementary Figure 5: The probability of hash collision estimated, using simulations, for different seeding methods with $n = 30$ and $k = 18, 21, 24, 27$. Only the results for $k = 18$ is reported for SubseqHash with a fully random order.

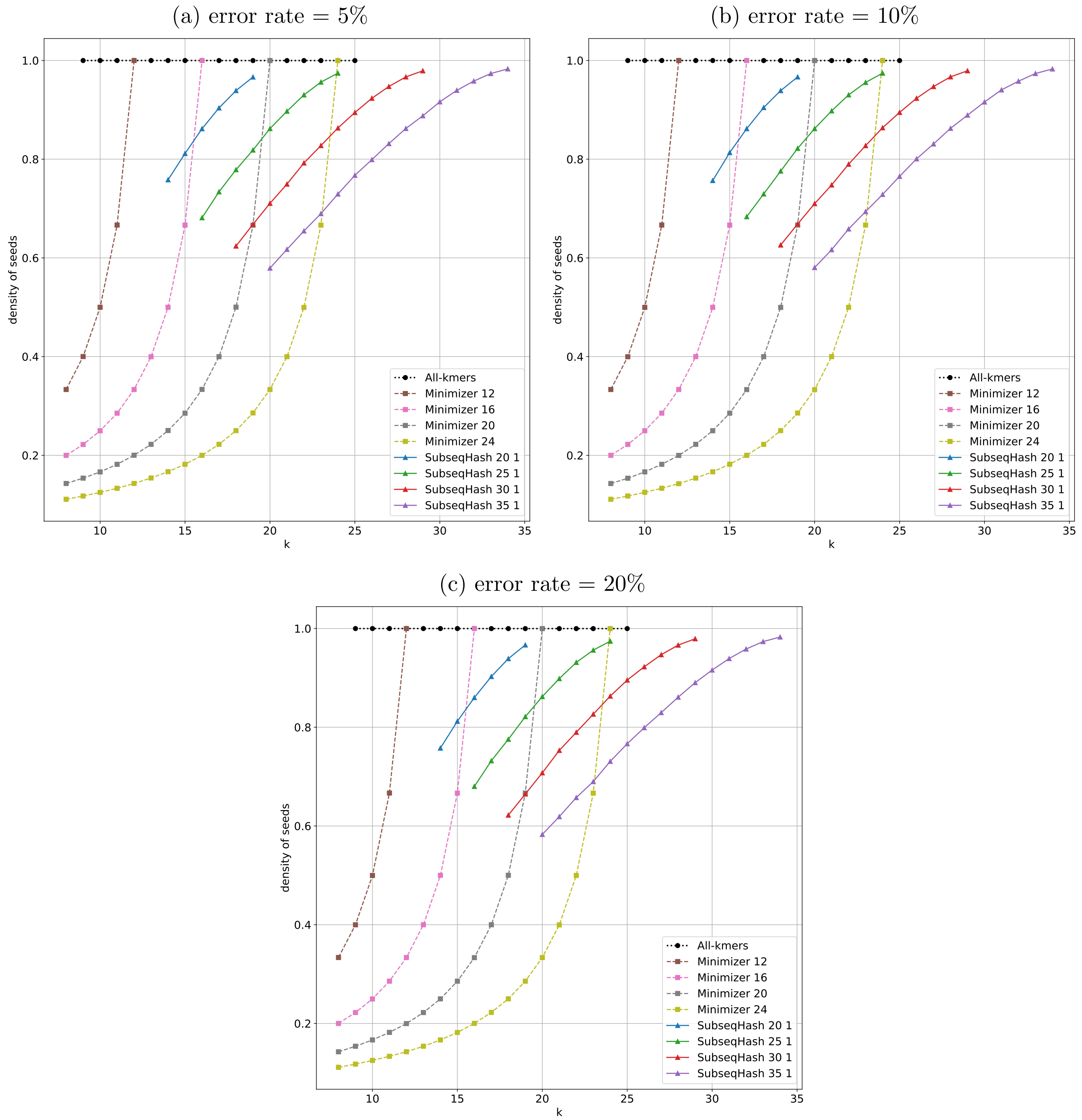

Supplementary Figure 6: The density of seeds for different seeding methods on simulated sequences with varying error rates (3 subfigures).

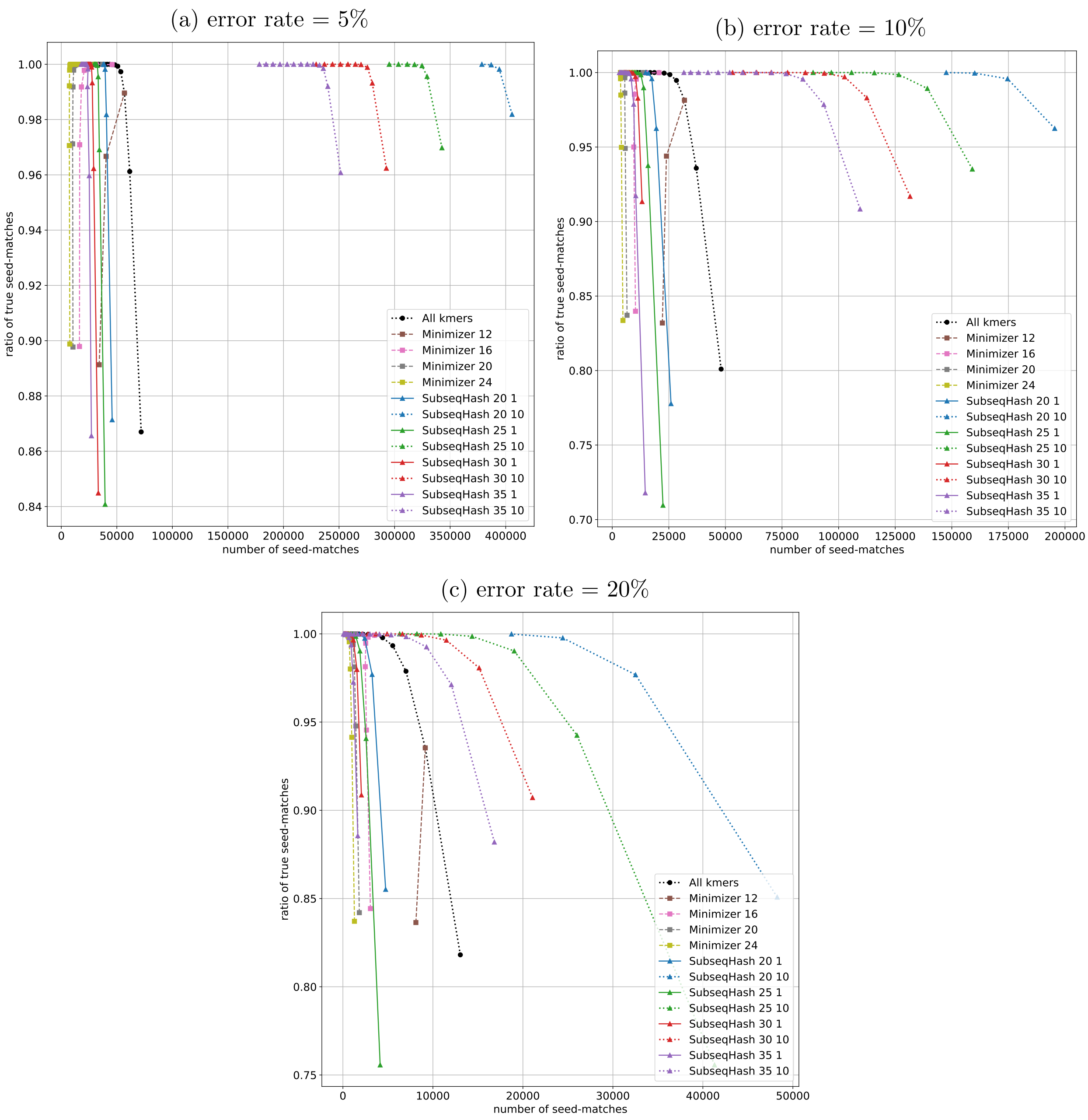

Supplementary Figure 7: The number of seed-matches and the ratio of true seed-matches for different seeding methods on simulated sequences with varying error rates (3 subfigures). Figures are cropped to only show the portion with high ratio of true seed-matches ($\geq 70\%$).

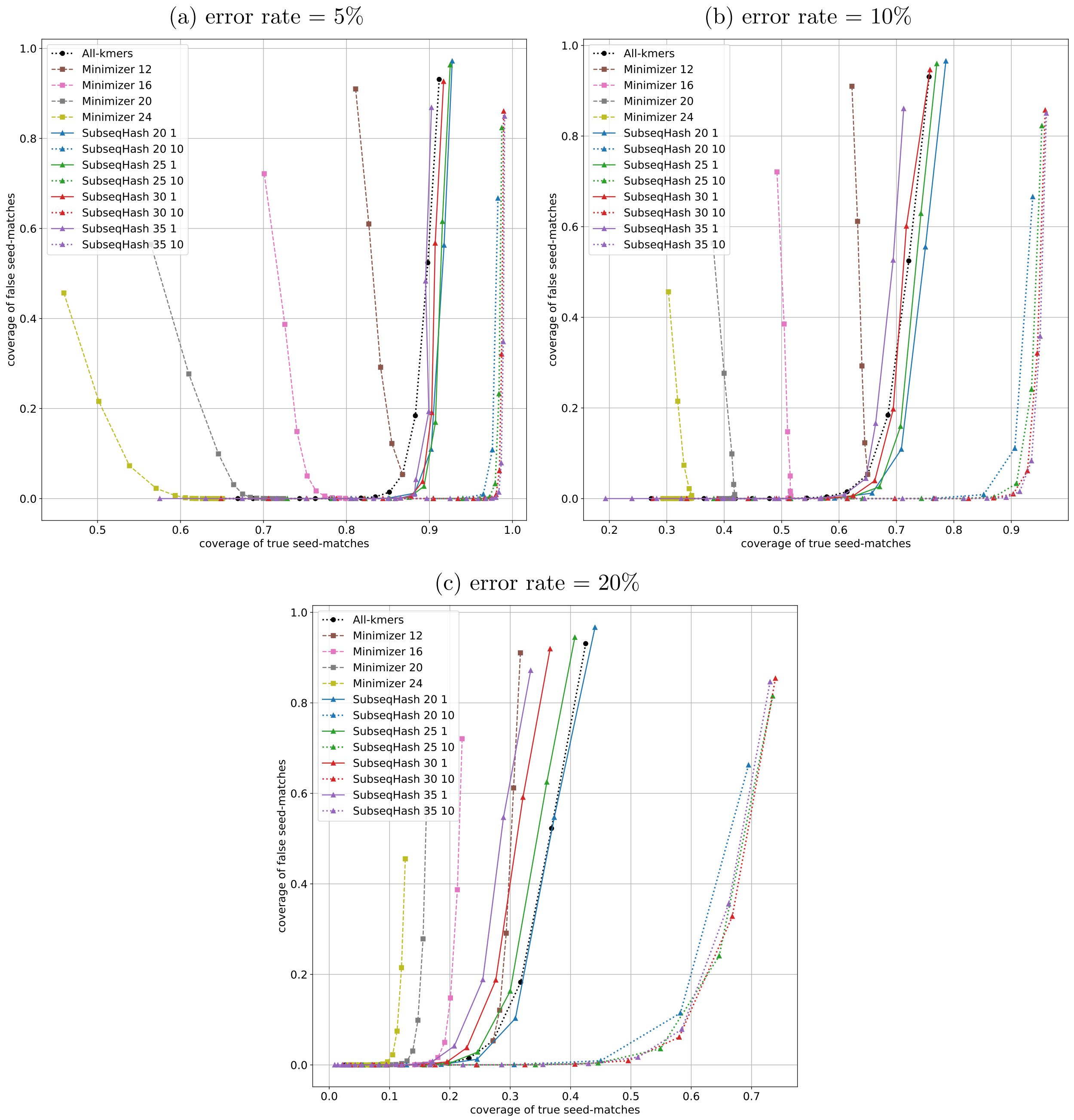

Supplementary Figure 8: The coverages of true and false seed-matches for different seeding methods on simulated sequences with varying error rates (3 subfigures).

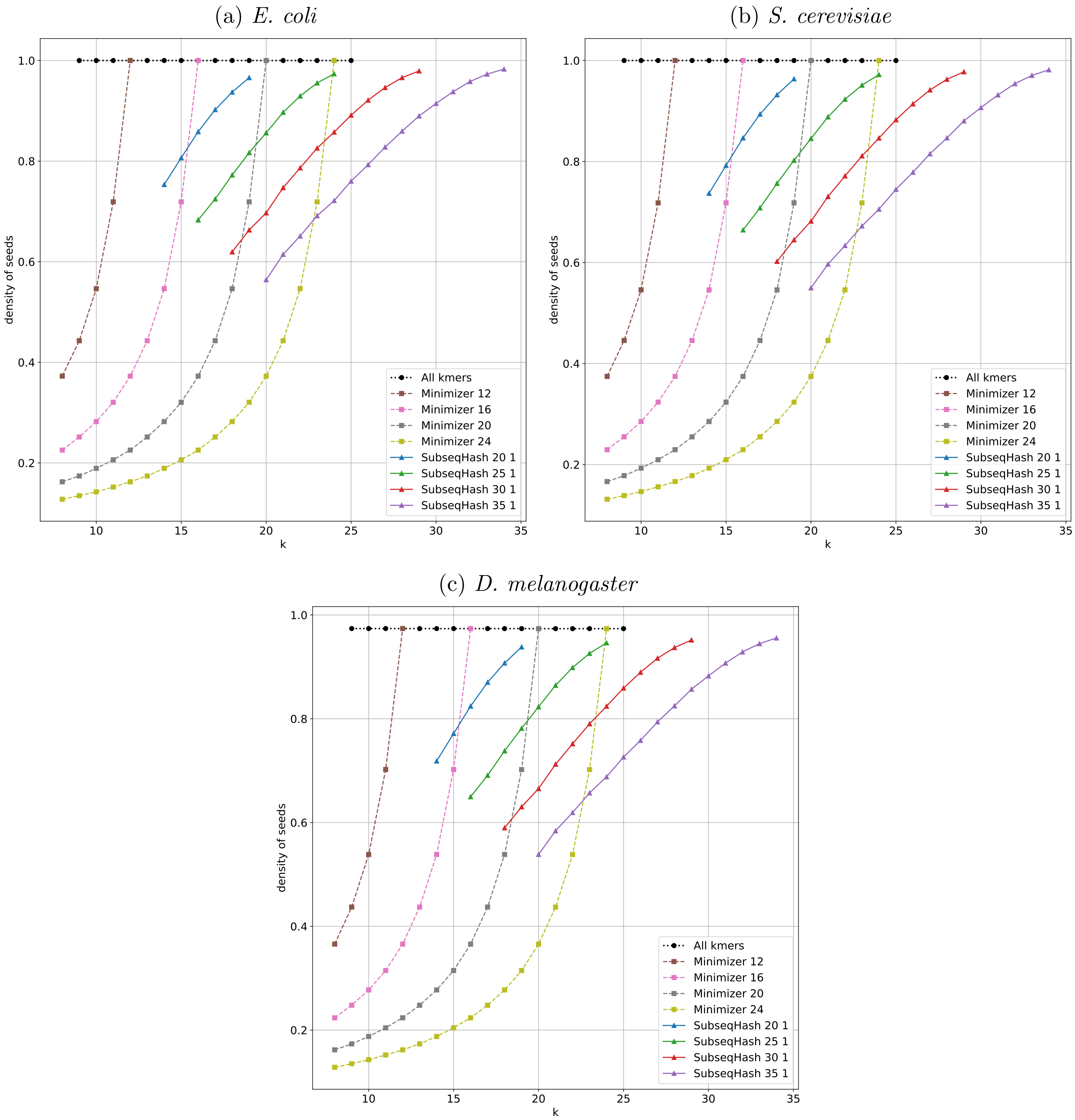

Supplementary Figure 9: The density of seeds for different seeding methods generated from reference genome of *E. coli*, *S. cerevisiae*, and *D. melanogaster*.

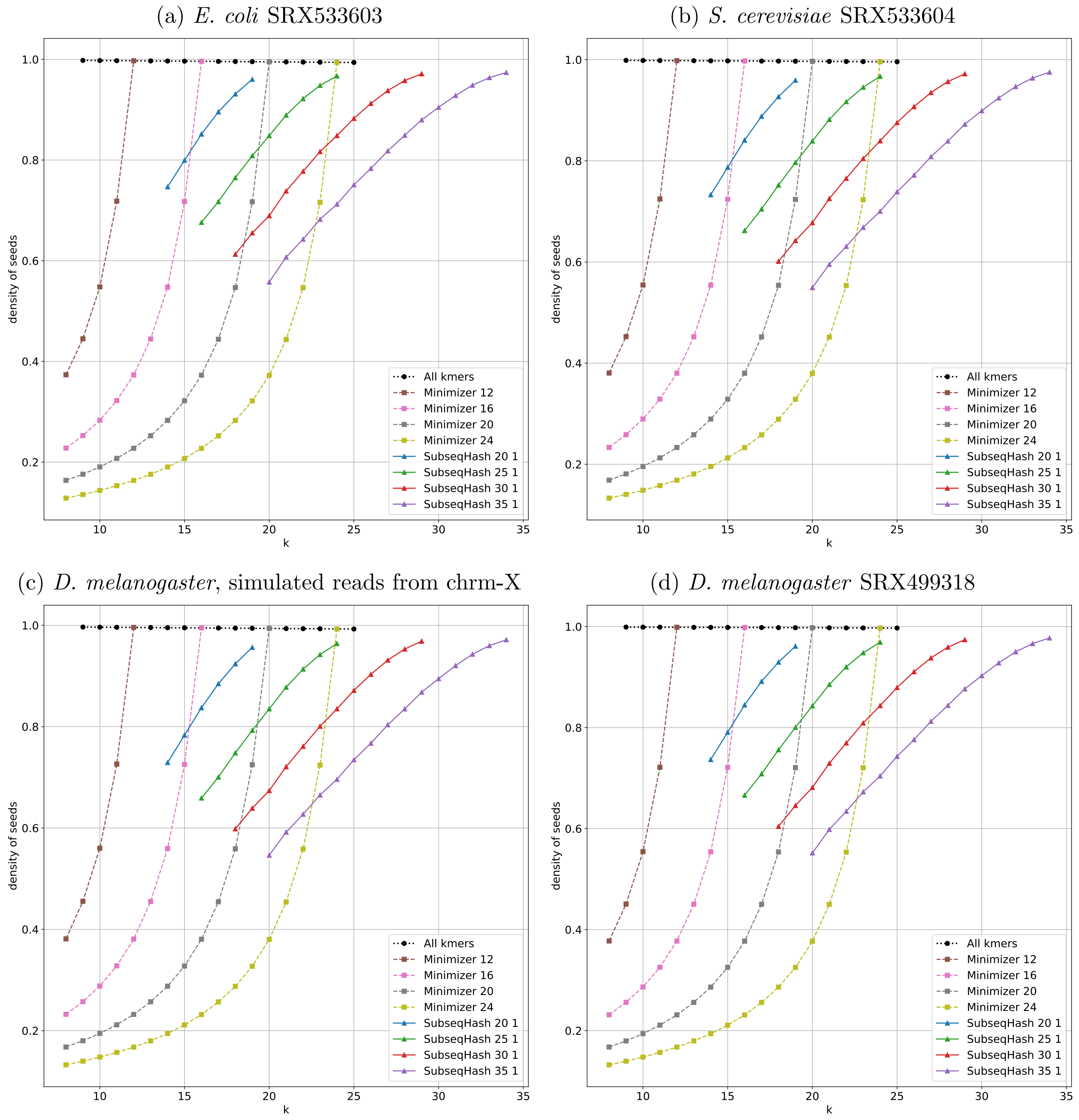

Supplementary Figure 10: The density of seeds for different seeding methods generated from three biological and one simulated datasets.

(a) *E. coli* SRX533603

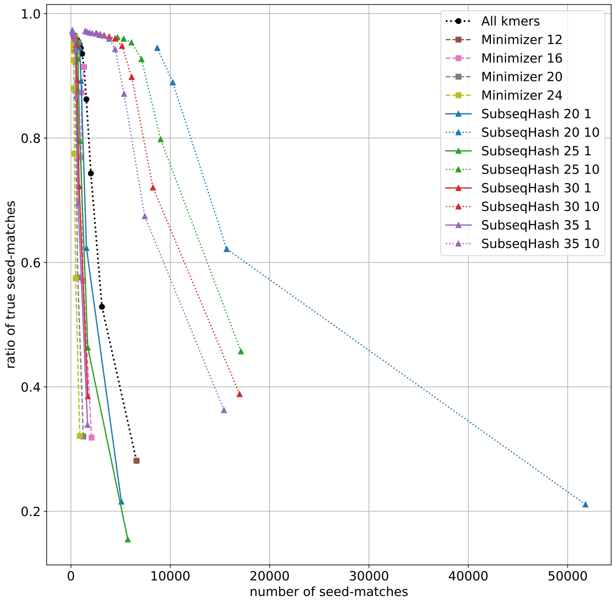

(b) *S. cerevisiae* SRX533604

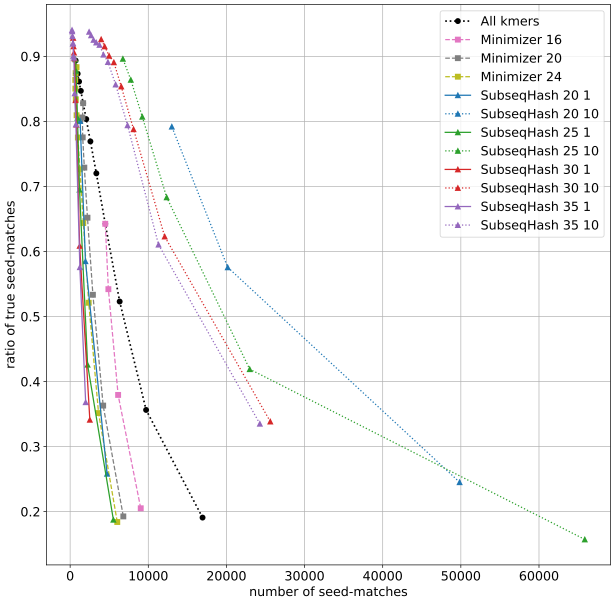

(c) *D. melanogaster*, simulated reads from chrm-X

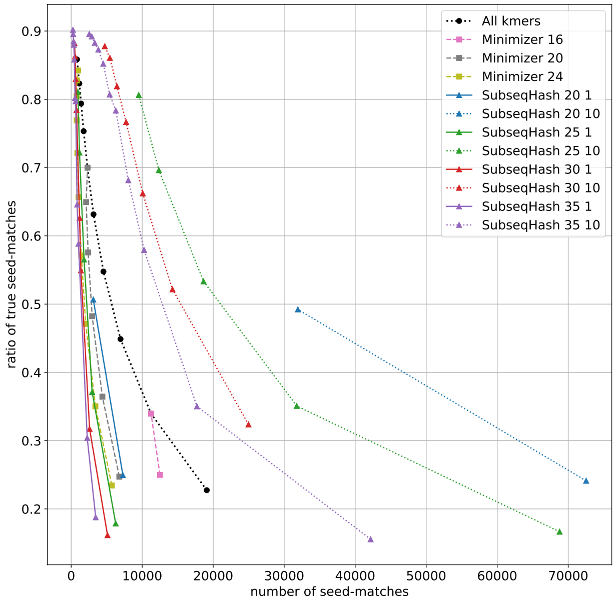

Supplementary Figure 11: The number of seed-matches and the ratio of true seed-matches for different seeding methods evaluated on 3 datasets (3 subfigures). Figures are cropped to only show the portion with high ratio of true seed-matches ($\geq 15\%$).

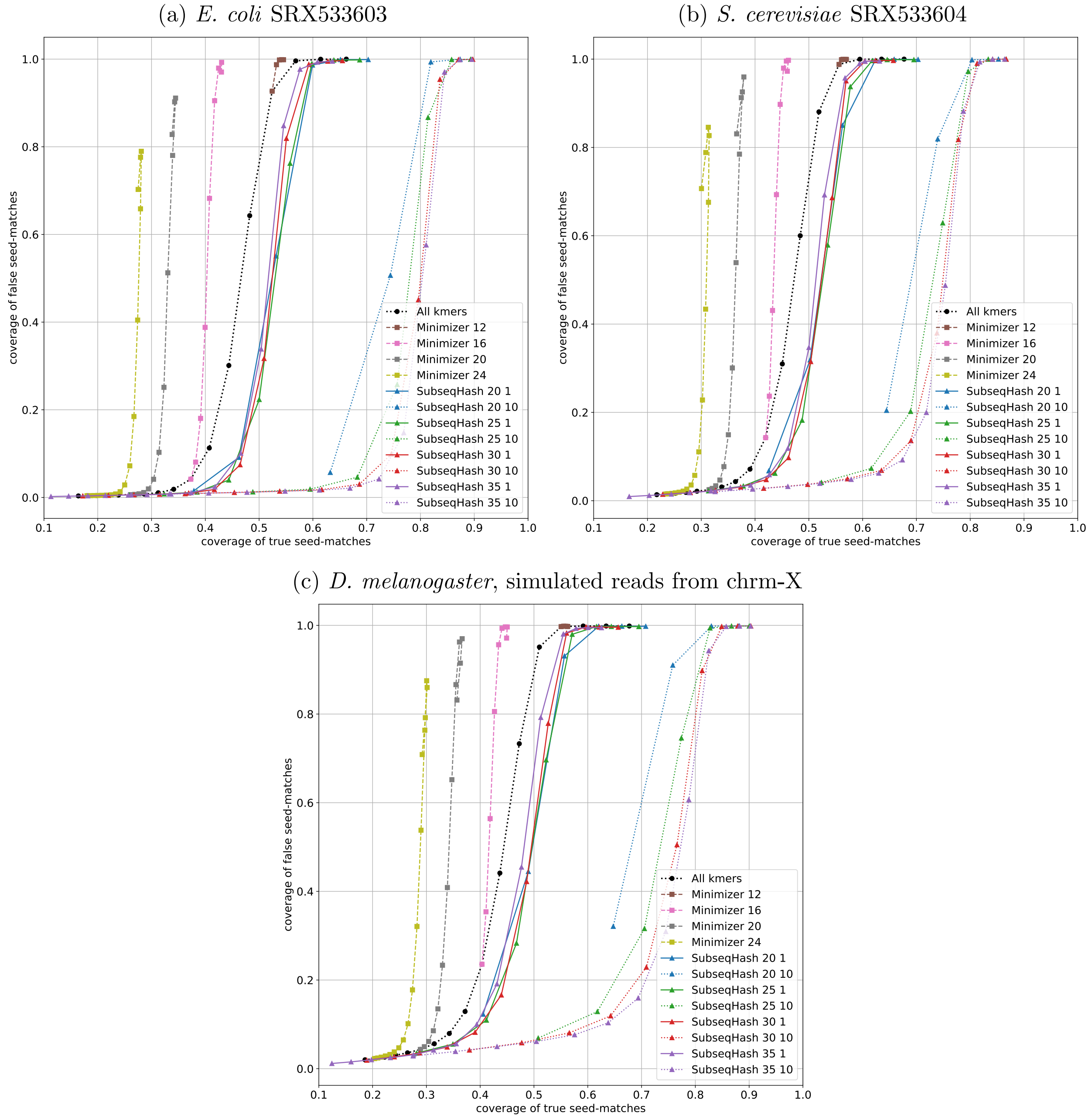

Supplementary Figure 12: The coverages of true and false seed-matches for different seeding methods on 3 datasets (3 subfigures).

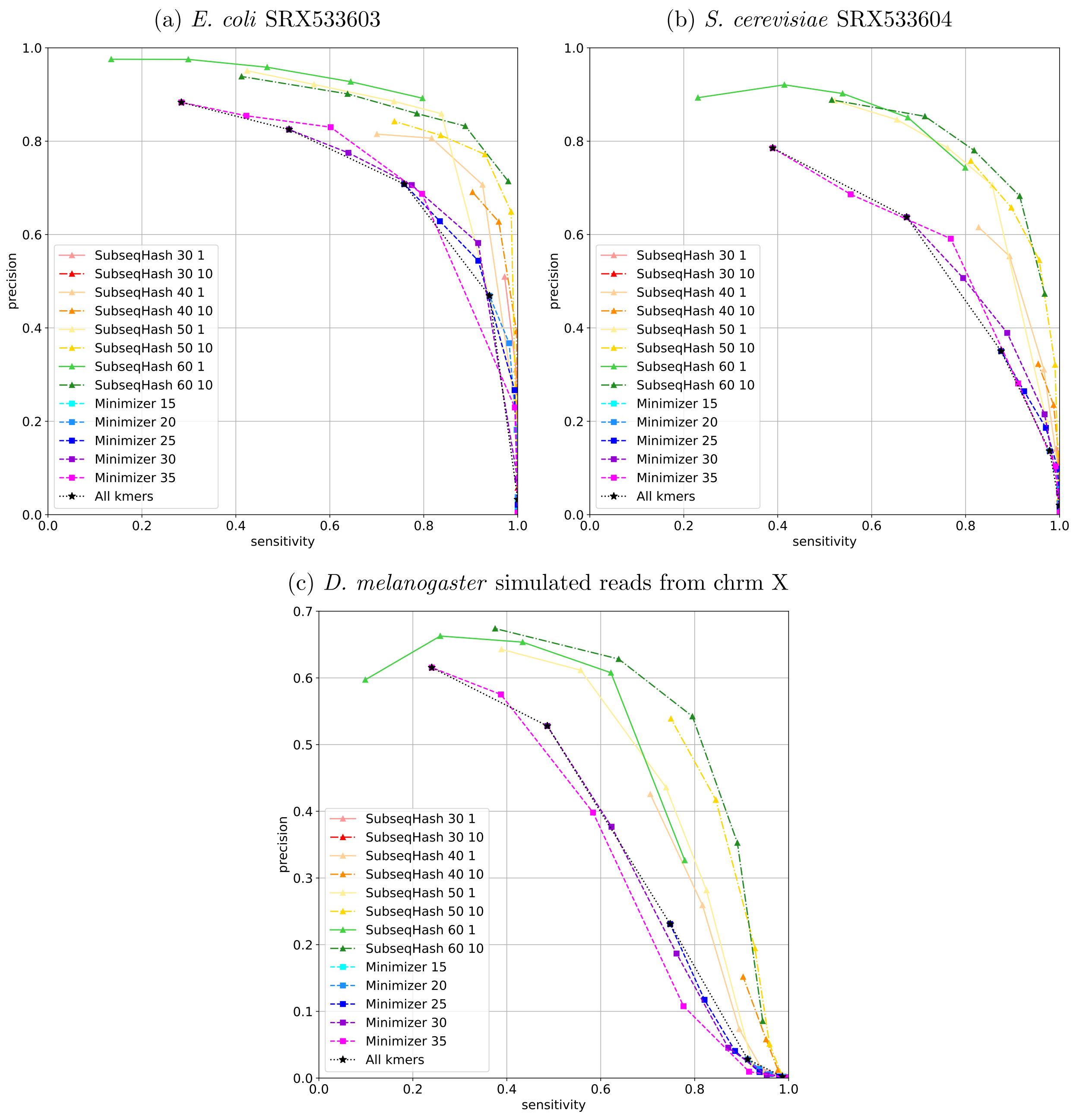

Supplementary Figure 13: Overlap detection results on reads sampled from 3 datasets.

Supplementary Table 2: This table lists the numbers of seed-matches ("number") and the ratio of true seed-matches ("ratio") of SubseqHash with different n and k. The results are from the pairwise sequence alignment described in Section 3.4 on four simulation datasets with different error rates. Part of this table (ratio ≥ 0.70) is used to draw Fig. 5 and Supplementary Figure 7.

Method	n	k	error rate 5%		error rate 10%		error rate 15%		error rate 20%	
			number	ratio	number	ratio	number	ratio	number	ratio
SubseqHash	20	14	77474.1	0.51	58270.5	0.37	46856.4	0.25	42648.4	0.15
		15	45788.4	0.87	25970.1	0.78	15859.5	0.64	10769.5	0.48
		16	40604.6	0.98	19473.3	0.96	9433.9	0.93	4749.4	0.86
		17	39448.8	1.00	17484.4	1.00	7499.2	0.99	3241.2	0.98
		18	38672.2	1.00	16029.5	1.00	6341.4	1.00	2429.6	1.00
		19	37840.5	1.00	14772.3	1.00	5417.0	1.00	1878.2	1.00
	25	16	67557.0	0.50	50404.2	0.34	39713.3	0.22	36105.9	0.14
		17	39464.0	0.84	22416.6	0.71	14593.9	0.55	10495.4	0.38
		18	34268.4	0.97	15826.7	0.94	7627.2	0.87	4138.6	0.76
		19	33211.2	1.00	13856.3	0.99	5859.7	0.97	2574.5	0.94
		20	32438.3	1.00	12672.9	1.00	4879.4	1.00	1890.2	0.99
		21	31941.9	1.00	11593.3	1.00	4095.9	1.00	1433.7	1.00
		22	31142.5	1.00	10573.8	1.00	3430.9	1.00	1090.9	1.00
		23	30394.2	1.00	9686.8	1.00	2888.6	1.00	826.8	1.00
		24	29502.0	1.00	8884.9	1.00	2468.6	1.00	629.9	1.00
	30	18	54758.6	0.53	42168.6	0.34	29987.2	0.23	27138.5	0.13
		19	33333.7	0.84	18164.0	0.69	10708.8	0.55	8512.0	0.35
		20	29136.1	0.96	13180.9	0.91	6379.7	0.84	3473.0	0.69
		21	27858.1	0.99	11327.9	0.98	4641.4	0.96	2055.8	0.91
		22	27242.2	1.00	10258.3	1.00	3893.1	0.99	1556.5	0.98
		23	26977.7	1.00	9277.5	1.00	3267.6	1.00	1160.4	1.00
		24	26343.5	1.00	8527.0	1.00	2690.4	1.00	861.1	1.00
		25	25803.2	1.00	7738.1	1.00	2260.3	1.00	661.8	1.00
		26	25018.3	1.00	7061.9	1.00	1897.2	1.00	499.1	1.00
		27	24376.6	1.00	6383.8	1.00	1577.9	1.00	372.0	1.00
		28	23697.1	1.00	5827.5	1.00	1326.9	1.00	275.6	1.00
		29	22940.9	1.00	5311.5	1.00	1125.7	1.00	211.7	1.00
	35	20	41523.2	0.59	27550.4	0.41	22412.2	0.24	19721.9	0.14
		21	27118.8	0.87	14591.3	0.72	9095.3	0.52	6720.4	0.34
		22	25162.1	0.96	10407.7	0.92	4979.4	0.81	2885.3	0.66
		23	23555.1	0.99	9477.3	0.98	3797.1	0.94	1651.2	0.89
		24	24052.5	1.00	8377.0	1.00	3109.0	0.99	1157.2	0.97
		25	23129.7	1.00	7611.5	1.00	2606.5	1.00	908.8	0.99
		26	22856.1	1.00	7053.1	1.00	2207.6	1.00	659.5	1.00
		27	22282.7	1.00	6329.8	1.00	1839.2	1.00	538.3	1.00
		28	21613.3	1.00	5714.7	1.00	1525.5	1.00	402.3	1.00
		29	20927.8	1.00	5188.2	1.00	1252.4	1.00	294.1	1.00
		30	20219.3	1.00	4651.8	1.00	1046.9	1.00	227.7	1.00
		31	19633.8	1.00	4216.2	1.00	871.0	1.00	152.8	1.00
		32	19018.8	1.00	3810.9	1.00	729.7	1.00	122.2	1.00
		33	18436.2	1.00	3462.1	1.00	626.9	1.00	88.2	1.00
		34	17827.6	1.00	3159.6	1.00	530.5	1.00	67.9	1.00

Supplementary Table 3: The table lists the numbers of seed-matches ("number") and the ratio of true seed-matches ("ratio") of repeating SubseqHash for 10 times with different n and k. The results are from the pairwise sequence alignment described in Section 3.4 on four simulation datasets with different error rates. Part of this table (ratio ≥ 0.70) is used to draw Fig. 5 and Supplementary Figure 7.

Method	n	k	error rate 5%		error rate 10%		error rate 15%		error rate 20%	
			number	ratio	number	ratio	number	ratio	number	ratio
SubseqHash (repeating 10 times)	20	16	405680.8	0.98	195457.9	0.96	94643.7	0.92	48257.1	0.85
		17	394265.9	1.00	174637.3	1.00	75255.7	0.99	32520.0	0.98
		18	386939.6	1.00	160123.2	1.00	63547.2	1.00	24431.4	1.00
		19	378544.9	1.00	147540.0	1.00	54187.1	1.00	18731.9	1.00
	25	18	342610.6	0.97	159062.1	0.94	77561.9	0.87	41295.0	0.76
		19	329215.9	1.00	139205.4	0.99	58822.9	0.98	26009.9	0.94
		20	324693.3	1.00	126591.0	1.00	48651.2	1.00	19037.3	0.99
		21	317957.7	1.00	115788.1	1.00	40966.3	1.00	14355.8	1.00
		22	311435.1	1.00	105724.2	1.00	34397.6	1.00	10879.9	1.00
		23	303495.4	1.00	96798.5	1.00	28953.5	1.00	8226.4	1.00
		24	295151.4	1.00	88724.4	1.00	24672.9	1.00	6301.8	1.00
	30	20	292559.4	0.96	131554.7	0.92	62840.7	0.83	34616.8	0.69
		21	279921.4	0.99	112461.4	0.98	46625.8	0.96	21070.2	0.91
		22	275518.8	1.00	102643.2	1.00	38465.5	0.99	15137.9	0.98
		23	269784.7	1.00	93686.2	1.00	32390.1	1.00	11500.9	1.00
		24	264714.0	1.00	85330.0	1.00	27163.2	1.00	8715.6	1.00
		25	257998.1	1.00	77462.9	1.00	22678.1	1.00	6611.9	1.00
		26	251244.7	1.00	70238.4	1.00	18904.6	1.00	4908.4	1.00
		27	244137.5	1.00	63789.5	1.00	15734.3	1.00	3681.7	1.00
		28	236888.9	1.00	58037.9	1.00	13253.0	1.00	2786.0	1.00
		29	229416.0	1.00	53147.6	1.00	11288.6	1.00	2123.1	1.00
	35	22	251457.4	0.96	109481.4	0.91	51081.9	0.82	28546.5	0.66
		23	239902.7	0.99	93475.0	0.98	37922.6	0.95	16826.4	0.88
		24	235866.7	1.00	84224.0	1.00	31082.2	0.99	12050.5	0.97
		25	232408.1	1.00	76802.6	1.00	26015.4	1.00	9291.4	0.99
		26	226653.1	1.00	70403.7	1.00	21966.5	1.00	7027.7	1.00
		27	221466.0	1.00	63496.2	1.00	18423.9	1.00	5349.9	1.00
		28	215687.5	1.00	57577.7	1.00	15270.2	1.00	4053.1	1.00
		29	209136.4	1.00	51868.8	1.00	12792.3	1.00	2994.8	1.00
		30	203279.5	1.00	46761.7	1.00	10566.7	1.00	2226.2	1.00
		31	196830.7	1.00	42278.2	1.00	8791.2	1.00	1643.4	1.00
		32	190531.3	1.00	38108.8	1.00	7368.5	1.00	1206.3	1.00
		33	184317.6	1.00	34619.8	1.00	6228.8	1.00	900.6	1.00
		34	178289.5	1.00	31643.5	1.00	5306.9	1.00	685.4	1.00

Supplementary Table 4: The table lists the numbers of seed-matches ("number") and the ratio of true seed-matches ("ratio") of kmer with different k. The results are from the pairwise sequence alignment described in Section 3.4 on four simulation datasets with different error rates. Part of this table (ratio ≥ 0.70) is used to draw Fig. 5 and Supplementary Figure 7.

Method	k	error rate 5%		error rate 10%		error rate 15%		error rate 20%	
		number	ratio	number	ratio	number	ratio	number	ratio
kmer	9	103874.2	0.63	80977.7	0.53	65006.6	0.41	54838.9	0.30
	10	71897.3	0.87	48107.3	0.80	32421.1	0.70	22846.2	0.58
	11	61600.2	0.96	37121.8	0.94	21862.5	0.89	13047.3	0.82
	12	56838.3	0.99	31908.3	0.98	17188.8	0.96	9150.9	0.94
	13	53568.4	1.00	28371.3	0.99	14288.1	0.99	7009.9	0.98
	14	50773.2	1.00	25467.4	1.00	12095.0	1.00	5533.7	0.99
	15	48194.0	1.00	22928.1	1.00	10292.2	1.00	4411.3	1.00
	16	45759.9	1.00	20659.7	1.00	8772.0	1.00	3517.9	1.00
	17	43458.8	1.00	18617.0	1.00	7480.5	1.00	2805.1	1.00
	18	41274.5	1.00	16779.1	1.00	6374.4	1.00	2236.2	1.00
	19	39200.0	1.00	15120.0	1.00	5426.4	1.00	1785.7	1.00
	20	37232.7	1.00	13624.3	1.00	4617.0	1.00	1426.9	1.00
	21	35368.2	1.00	12281.9	1.00	3928.3	1.00	1138.8	1.00
	22	33597.4	1.00	11069.1	1.00	3345.0	1.00	911.9	1.00
	23	31914.5	1.00	9980.5	1.00	2851.9	1.00	730.9	1.00
	24	30316.0	1.00	8998.8	1.00	2432.6	1.00	585.6	1.00
	25	28798.4	1.00	8113.9	1.00	2074.1	1.00	469.1	1.00

Supplementary Table 5: The table lists the numbers of seed-matches ("number") and the ratio of true seed-matches ("ratio") of minimizer with different n and k. The results are from the pairwise sequence alignment described in Section 3.4 on four simulation datasets with different error rates. Part of this table (ratio ≥ 0.70) is used to draw Fig. 5 and Supplementary Figure 7.

Method	n	k	error rate 5%		error rate 10%		error rate 15%		error rate 20%	
			number	ratio	number	ratio	number	ratio	number	ratio
Minimizer	12	8	58869.2	0.38	51313.3	0.28	46072.1	0.20	42716.8	0.14
		9	36747.0	0.69	27378.9	0.59	21141.1	0.46	17264.7	0.34
		10	34132.8	0.89	22135.6	0.83	14422.7	0.74	9802.5	0.62
		11	40328.1	0.97	23938.8	0.94	13860.6	0.90	8109.2	0.84
	16	8	33593.8	0.38	28973.7	0.29	25938.4	0.20	23969.6	0.14
		9	19446.1	0.70	14212.6	0.59	10857.0	0.46	8841.2	0.34
		10	16354.3	0.90	10277.4	0.84	6565.3	0.75	4380.4	0.62
		11	16548.7	0.97	9510.3	0.95	5360.6	0.91	3056.7	0.84
		12	18116.6	0.99	9784.6	0.99	5094.9	0.97	2619.2	0.95
		13	20695.8	1.00	10644.4	1.00	5211.5	0.99	2486.3	0.98
		14	24780.7	1.00	12173.4	1.00	5660.9	1.00	2535.0	0.99
		15	31739.7	1.00	14906.1	1.00	6612.0	1.00	2798.0	1.00
	20	8	23450.8	0.38	20124.3	0.28	18066.2	0.20	16653.4	0.13
		9	13047.5	0.70	9459.1	0.59	7212.5	0.46	5918.7	0.33
		10	10510.3	0.90	6549.1	0.84	4126.6	0.74	2782.6	0.62
		11	10197.7	0.97	5734.9	0.95	3205.5	0.91	1824.9	0.84
		12	10530.8	0.99	5545.8	0.99	2846.9	0.97	1441.9	0.95
		13	11125.6	1.00	5567.2	1.00	2689.4	0.99	1271.3	0.98
		14	11989.1	1.00	5707.9	1.00	2596.8	1.00	1137.8	0.99
		15	13056.0	1.00	5966.8	1.00	2574.4	1.00	1061.5	1.00
		16	14633.6	1.00	6366.3	1.00	2613.6	1.00	1013.9	1.00
		17	16799.3	1.00	6998.3	1.00	2714.3	1.00	1001.0	1.00
		18	20203.9	1.00	8019.3	1.00	2992.2	1.00	1023.5	1.00
		19	25786.8	1.00	9831.5	1.00	3495.2	1.00	1131.9	1.00
	24	8	17876.8	0.38	15387.5	0.28	13730.8	0.20	12771.8	0.13
		9	9807.8	0.70	7065.6	0.58	5380.8	0.45	4405.3	0.33
		10	7669.0	0.90	4733.9	0.83	3035.2	0.73	2041.3	0.61
		11	7249.7	0.97	4047.3	0.95	2246.0	0.91	1282.2	0.84
		12	7276.1	0.99	3790.3	0.98	1934.4	0.97	985.7	0.94
		13	7486.8	1.00	3657.7	1.00	1740.9	0.99	819.2	0.98
		14	7744.5	1.00	3638.7	1.00	1633.2	1.00	701.5	1.00
		15	8043.3	1.00	3613.5	1.00	1544.2	1.00	631.2	1.00
		16	8496.5	1.00	3604.2	1.00	1457.3	1.00	558.5	1.00
		17	9035.3	1.00	3681.3	1.00	1398.7	1.00	499.9	1.00
		18	9709.0	1.00	3783.9	1.00	1375.3	1.00	462.2	1.00
		19	10652.2	1.00	3903.6	1.00	1364.0	1.00	429.4	1.00
		20	11906.2	1.00	4198.1	1.00	1384.4	1.00	406.9	1.00
		21	13685.3	1.00	4604.8	1.00	1432.7	1.00	406.8	1.00
		22	16401.9	1.00	5299.2	1.00	1571.4	1.00	415.6	1.00
		23	21000.5	1.00	6499.9	1.00	1839.4	1.00	466.4	1.00

Supplementary Table 6: The table lists running time in seconds of using SubseqHash with different n and k in seeding step. The results are from the pairwise sequence alignment described in Section 3.4 on four simulation datasets with different error rates.

Method	n	k	Running time			
			5%	10%	15%	20%
SubseqHash	20	14	157.2	157.0	157.0	158.0
		15	146.4	147.5	147.1	146.6
		16	124.6	124.0	123.7	124.4
		17	94.2	93.9	93.5	93.8
		18	71.0	70.6	71.2	73.4
		19	45.6	45.9	45.6	45.4
	25	16	241.1	238.9	238.2	236.7
		17	241.9	241.6	241.5	241.6
		18	233.3	234.6	232.4	233.6
		19	222.1	222.6	222.4	219.6
		20	197.5	196.7	198.1	196.5
		21	167.2	168.2	166.9	167.9
		22	134.4	134.6	134.5	134.0
		23	97.3	97.2	96.9	97.5
		24	56.7	56.9	56.5	55.4
	30	18	406.1	407.4	407.5	408.8
		19	355.1	352.6	352.7	354.9
		20	373.3	373.8	373.3	371.3
		21	357.2	358.4	356.6	355.2
		22	356.6	357.8	356.6	356.7
		23	304.5	305.3	306.2	305.6
		24	277.7	278.4	277.5	276.6
		25	241.9	246.5	246.0	246.2
		26	212.4	212.3	212.1	212.1
		27	160.8	161.1	161.3	161.0
		28	124.3	124.5	123.3	123.8
		29	73.6	73.1	74.2	74.2
	35	20	541.8	531.4	527.9	529.4
		21	537.0	538.7	538.8	536.7
		22	530.2	527.6	527.7	528.8
		23	526.4	532.3	525.5	504.4
		24	512.8	515.2	514.7	516.6
		25	504.9	503.9	503.4	503.1
		26	452.6	452.0	447.1	446.3
		27	435.2	433.3	433.8	432.9
		28	399.3	400.3	400.3	399.5
		29	357.1	356.4	356.5	354.3
		30	311.5	310.5	308.8	310.3
		31	255.0	252.6	252.5	252.0
		32	217.0	217.3	217.6	215.9
		33	142.4	142.8	142.9	142.5
		34	83.9	83.8	83.8	83.9

Supplementary Table 7: The table lists running time in seconds of Repeating SubseqHash for 10 times with different n and k in seeding step. The results are from the pairwise sequence alignment described in Section 3.4 on four simulation datasets with different error rates.

Method	n	k	Running time			
			5%	10%	15%	20%
SubseqHash (repeating 10 times)	20	16	1239.2	1242.7	1242.6	1244.0
		17	937.8	942.1	938.1	939.7
		18	743.4	742.9	742.5	741.2
		19	455.6	456.3	456.0	455.8
	25	18	2284.4	2260.8	2259.7	2327.0
		19	2117.4	2012.5	2000.8	1973.9
		20	1886.9	1777.6	1770.4	1772.2
		21	1645.7	1591.4	1586.4	1556.9
		22	1324.6	1271.4	1266.8	1265.3
		23	891.5	892.2	887.8	890.0
		24	557.1	558.1	552.1	536.2
	30	20	3609.8	3473.1	3440.2	3439.7
		21	3521.7	3527.9	3515.5	3478.2
		22	3196.3	3191.6	3195.7	3169.7
		23	2997.6	2939.6	2908.9	2901.6
		24	2922.7	2933.6	2920.0	2930.3
		25	2511.8	2468.7	2455.5	2369.0
		26	2113.2	2065.9	2069.0	2027.8
		27	1669.4	1628.6	1629.0	1619.7
		28	1155.8	1096.5	1090.5	1090.9
		29	626.5	629.5	623.9	622.5
	35	22	5166.9	5040.4	5047.8	4981.8
		23	5207.2	5197.5	5208.5	5196.3
		24	5160.3	5175.1	5163.1	5158.1
		25	4947.2	4957.9	4931.3	4941.2
		26	4339.7	4200.9	4201.1	4141.7
		27	4279.1	4206.0	3941.0	4010.2
		28	3713.6	3623.8	3630.5	3620.4
		29	3420.3	3300.6	3300.1	3291.2
		30	3099.8	3075.8	3048.0	3036.1
		31	2305.2	2251.2	2234.0	2238.6
		32	1869.6	1838.4	1834.1	1827.1
		33	1429.7	1434.7	1431.4	1432.1
		34	840.0	840.5	841.3	840.3

Supplementary Table 8: The table lists running time in seconds of using kmer with different k in seeding step. The results are from the pairwise sequence alignment described in Section 3.4 on four simulation datasets with different error rates.

Method	k	Running time			
		5%	10%	15%	20%
kmer	9	12.3	12.2	12.2	12.3
	10	11.6	11.6	12.0	12.3
	11	12.0	11.8	12.2	12.5
	12	12.1	12.0	12.4	12.7
	13	12.1	12.1	12.5	12.8
	14	12.7	12.7	12.7	12.9
	15	12.8	12.9	12.8	13.1
	16	12.2	12.1	12.2	12.3
	17	12.0	12.0	12.0	11.9
	18	13.6	13.7	13.7	13.7
	19	13.9	13.9	13.8	13.9
	20	14.1	14.0	14.0	14.0
	21	14.2	14.2	14.3	14.2
	22	14.4	14.3	14.3	14.4
	23	14.5	14.0	13.3	13.2
	24	14.6	14.0	13.5	13.5
	25	14.8	14.2	13.7	13.6

Supplementary Table 9: The table lists running time in seconds of using minimizer with different n and k in seeding step. The results are from the pairwise sequence alignment described in Section 3.4 on four simulation datasets with different error rates.

Method	n	k	Running time			
			5%	10%	15%	20%
Minimizer	12	8	4.4	4.5	4.5	4.4
		9	4.8	4.8	4.8	5.0
		10	5.6	5.6	5.6	5.7
		11	8.7	8.7	8.6	8.7
	16	8	3.0	3.0	3.0	3.0
		9	3.3	3.3	3.3	3.3
		10	3.6	3.6	3.6	3.6
		11	4.1	4.1	4.1	4.1
		12	4.7	4.7	4.7	4.7
		13	5.5	5.4	5.5	5.4
		14	6.2	6.2	6.2	6.2
		15	7.9	8.0	7.9	7.9
	20	8	2.4	2.4	2.4	2.4
		9	2.5	2.5	2.5	2.5
		10	2.8	2.7	2.7	2.7
		11	3.0	2.9	2.9	2.9
		12	3.1	3.1	3.1	3.1
		13	3.4	3.4	3.5	3.4
		14	3.4	3.3	3.4	3.3
		15	3.8	3.8	3.8	3.7
		16	4.8	4.8	4.8	4.8
		17	5.7	5.7	5.6	5.6
		18	7.2	7.2	7.2	7.2
		19	9.6	9.6	9.5	9.4
	24	8	2.1	2.1	2.1	2.1
		9	2.1	2.2	2.2	2.2
		10	2.2	2.3	2.2	2.3
		11	2.3	2.3	2.3	2.3
		12	2.5	2.5	2.5	2.4
		13	2.4	2.4	2.4	2.4
		14	2.5	2.6	2.5	2.6
		15	2.7	2.8	2.8	2.8
		16	3.1	3.2	3.2	3.1
		17	3.5	3.5	3.4	3.4
		18	3.6	3.7	3.6	3.6
		19	3.8	3.8	4.0	4.1
		20	5.2	5.2	5.2	5.2
		21	6.1	6.2	6.1	6.1
		22	7.5	7.5	7.5	7.4
		23	10.0	10.0	9.9	9.9

Supplementary Table 10: The table lists the numbers of seed-matches ("number") and the ratio of true seed-matches ("ratio") of SubseqHash with different n and k. The results are from the read mapping described in Section 3.5 on four datasets. Part of this table (ratio ≥ 0.15) is used to draw Fig. 7 and Supplementary Figure 11.

Method	n	k	E.coli		S.cerevisiae		Simulation		D.melanogaster	
			number	ratio	number	ratio	number	ratio	number	ratio
SubseqHash	20	14	158663.7	0.01	542563.7	0.00	1213503.4	0.00	9728455.7	0.00
		15	29747.5	0.04	131502.6	0.01	345242.2	0.00	2413021.7	0.00
		16	5077.8	0.22	24031.2	0.05	68340.6	0.02	422536.5	0.00
		17	1561.6	0.62	4722.8	0.26	15457.3	0.10	72964.2	0.02
		18	1021.3	0.89	1960.4	0.59	7284.1	0.25	21493.0	0.11
		19	869.0	0.95	1278.6	0.80	3120.1	0.51	8567.1	0.36
	25	16	131474.0	0.01	539151.3	0.00	1044891.5	0.00	8531299.8	0.00
		17	26976.4	0.04	94688.2	0.01	205185.8	0.01	1628726.4	0.00
		18	5726.1	0.15	26044.8	0.04	65095.9	0.02	467032.1	0.00
		19	1699.0	0.46	5533.2	0.19	13707.0	0.08	82991.1	0.02
		20	904.0	0.79	2233.7	0.43	6269.7	0.18	26128.3	0.05
		21	707.7	0.93	1200.3	0.70	2957.8	0.37	8950.7	0.19
		22	609.5	0.95	939.4	0.81	1817.0	0.57	5028.2	0.41
		23	532.1	0.96	756.4	0.88	1173.2	0.72	3057.6	0.60
		24	469.1	0.96	675.7	0.90	954.0	0.81	2443.5	0.70
	30	18	91264.9	0.01	284091.9	0.00	585928.2	0.00	4847207.3	0.00
		19	24250.0	0.04	66743.2	0.01	143034.3	0.01	1166062.9	0.00
		20	6040.0	0.12	23744.4	0.04	51843.4	0.02	411987.0	0.00
		21	1697.2	0.38	5728.6	0.15	13514.4	0.06	86811.5	0.01
		22	826.0	0.72	2519.9	0.34	5120.4	0.16	28041.5	0.04
		23	616.8	0.89	1217.9	0.61	2597.9	0.32	10120.3	0.12
		24	519.8	0.95	802.5	0.79	1367.4	0.55	3980.8	0.34
		25	449.2	0.96	690.6	0.83	1185.9	0.63	3175.6	0.49
		26	383.2	0.96	554.8	0.90	751.2	0.78	1859.4	0.68
		27	333.8	0.97	495.6	0.91	636.1	0.83	1564.0	0.73
		28	292.7	0.97	443.6	0.92	548.7	0.86	1334.9	0.77
		29	257.7	0.97	397.0	0.93	472.8	0.88	1099.8	0.80
	35	20	71997.1	0.01	219018.3	0.00	426294.2	0.00	3704395.7	0.00
		21	19771.2	0.04	52433.9	0.01	116124.3	0.01	936107.5	0.00
		22	4984.9	0.13	24142.6	0.03	43866.4	0.02	348312.4	0.00
		23	1674.0	0.34	7706.1	0.11	15676.4	0.05	116434.6	0.01
		24	730.3	0.70	1985.1	0.37	3454.3	0.19	19624.5	0.05
		25	543.3	0.87	1243.3	0.58	2263.2	0.30	9739.2	0.11
		26	440.6	0.94	734.4	0.80	1028.4	0.59	3347.8	0.32
		27	377.6	0.96	575.6	0.84	840.3	0.65	2252.5	0.47
		28	330.0	0.97	483.3	0.90	628.1	0.80	1574.4	0.66
		29	283.9	0.97	423.1	0.90	550.0	0.80	1343.5	0.69
		30	248.3	0.97	373.6	0.92	450.3	0.86	1065.1	0.77
		31	212.6	0.97	335.7	0.92	380.6	0.88	900.3	0.78
		32	187.1	0.97	299.1	0.93	336.1	0.88	765.7	0.80
		33	163.3	0.97	266.5	0.94	290.4	0.90	652.6	0.81
		34	144.4	0.97	243.2	0.94	255.0	0.90	564.6	0.83

Supplementary Table 11: The table lists the numbers of seed-matches ("number") and the ratio of true seed-matches ("ratio") of SubseqHash with repetition using different n and k. The results are from the read mapping described in Section 3.5 on four datasets. Part of this table (ratio ≥ 0.15) is used to draw Fig. 7 and Supplementary Figure 11.

Method	n	k	E.coli		S.cerevisiae		Simulation		D.melanogaster	
			number	ratio	number	ratio	number	ratio	number	ratio
SubseqHash (repeating 10 times)	20	16	51796.1	0.21	235032.8	0.06	706524.3	0.02	4263914.6	0.00
		17	15680.2	0.62	49840.2	0.25	175784.1	0.09	789897.1	0.02
		18	10254.7	0.89	20166.7	0.58	72523.9	0.24	219314.7	0.11
		19	8692.7	0.94	13011.2	0.79	31928.3	0.49	88357.6	0.35
	25	18	60951.2	0.15	261056.3	0.04	636805.3	0.02	4507959.8	0.00
		19	17110.7	0.46	65860.1	0.16	177058.3	0.06	1030530.0	0.01
		20	9028.7	0.80	22980.3	0.42	68785.9	0.17	276696.8	0.05
		21	7105.5	0.93	12353.3	0.68	31765.4	0.35	96464.9	0.18
		22	6089.7	0.95	9233.2	0.81	18645.6	0.53	49861.1	0.39
		23	5319.0	0.96	7763.7	0.86	12347.4	0.70	32374.9	0.58
		24	4687.8	0.96	6750.2	0.90	9517.2	0.81	24600.9	0.70
	30	20	57649.7	0.13	218719.2	0.04	476552.5	0.02	3597864.4	0.00
		21	16975.5	0.39	64734.7	0.14	144925.2	0.06	958614.7	0.01
		22	8256.8	0.72	25613.5	0.34	61906.2	0.14	304644.7	0.04
		23	6118.2	0.90	12099.2	0.62	24971.2	0.32	92370.5	0.13
		24	5148.9	0.95	8114.3	0.79	14272.4	0.52	41935.8	0.32
		25	4447.4	0.96	6540.5	0.85	10080.5	0.66	26226.8	0.52
		26	3851.8	0.96	5595.9	0.89	7739.4	0.77	19389.9	0.65
		27	3347.1	0.97	4989.3	0.90	6445.7	0.82	15823.9	0.72
		28	2922.8	0.97	4416.7	0.92	5451.4	0.86	12938.4	0.77
		29	2583.5	0.97	3970.0	0.93	4734.0	0.88	10941.1	0.80
	35	22	48417.2	0.13	205291.3	0.04	434053.3	0.02	3250099.1	0.00
		23	15403.4	0.36	60861.1	0.13	116700.4	0.06	833709.6	0.01
		24	7433.8	0.67	24278.9	0.34	42176.1	0.16	251008.2	0.04
		25	5354.4	0.87	11310.4	0.61	17714.2	0.35	78482.8	0.12
		26	4447.2	0.94	7344.5	0.79	10271.3	0.58	33643.5	0.31
		27	3852.8	0.96	5821.2	0.86	8046.6	0.68	21995.4	0.49
		28	3307.3	0.96	4836.4	0.89	6287.3	0.78	16078.1	0.64
		29	2876.0	0.97	4262.1	0.90	5422.6	0.81	13069.3	0.71
		30	2487.1	0.97	3755.6	0.92	4521.8	0.85	10729.1	0.76
		31	2144.7	0.97	3338.3	0.92	3829.7	0.87	8913.7	0.78
		32	1867.6	0.97	2992.2	0.93	3325.4	0.88	7605.3	0.80
		33	1631.4	0.97	2679.8	0.93	2900.2	0.89	6526.4	0.81
		34	1445.3	0.97	2428.1	0.94	2551.1	0.90	5655.2	0.82

Supplementary Table 12: The table lists the numbers of seed-matches ("number") and the ratio of true seed-matches ("ratio") of kmer with different k. The results are from the read mapping described in Section 3.5 on four datasets. Part of this table (ratio ≥ 0.15) is used to draw Fig. 7 and Supplementary Figure 11.

Method	k	E.coli		S.cerevisiae		Simulation		D.melanogaster	
		number	ratio	number	ratio	number	ratio	number	ratio
kmer	9	227902.3	0.01	764618.3	0.00	2165197.1	0.00	13919740.1	0.00
	10	63162.0	0.04	240974.2	0.01	772910.2	0.00	4427855.7	0.00
	11	18758.6	0.11	84768.5	0.03	305838.1	0.01	1544052.1	0.00
	12	6602.1	0.28	34718.5	0.08	134431.6	0.03	611062.4	0.01
	13	3124.0	0.53	16945.3	0.19	65100.4	0.06	279691.4	0.02
	14	2009.4	0.74	9722.1	0.36	34169.9	0.13	146527.1	0.04
	15	1558.4	0.86	6338.1	0.52	19095.9	0.23	85092.1	0.09
	16	1309.0	0.91	4493.8	0.64	11251.7	0.34	53029.9	0.18
	17	1132.0	0.94	3353.7	0.72	6951.5	0.45	34959.3	0.29
	18	989.4	0.94	2593.9	0.77	4547.8	0.55	24139.9	0.40
	19	868.7	0.95	2061.6	0.80	3147.8	0.63	17273.4	0.49
	20	764.2	0.95	1665.8	0.83	2300.1	0.70	12726.4	0.57
	21	673.3	0.96	1369.8	0.85	1761.0	0.75	9569.8	0.63
	22	593.6	0.96	1142.4	0.86	1404.3	0.79	7313.7	0.67
	23	523.8	0.96	961.0	0.87	1153.0	0.82	5674.8	0.70
	24	462.7	0.96	817.6	0.88	966.3	0.84	4466.0	0.73
	25	409.0	0.96	701.4	0.89	822.4	0.86	3556.9	0.75

Supplementary Table 13: The table lists the numbers of seed-matches ("number") and the ratio of true seed-matches ("ratio") of minimizer with different n and k. The results are from the read mapping described in Section 3.5 on four datasets. Part of this table (ratio ≥ 0.15) is used to draw Fig. 7 and Supplementary Figure 11.

Method	n	k	E.coli		S.cerevisiae		Simulation		D.melanogaster	
			number	ratio	number	ratio	number	ratio	number	ratio
Minimizer	12	8	249546.7	0.00	904468.7	0.00	2352730.5	0.00	16017975.8	0.00
		9	81389.4	0.01	313513.3	0.00	907971.8	0.00	5685240.6	0.00
		10	28327.9	0.05	121015.3	0.01	393496.6	0.00	2201461.1	0.00
		11	11451.3	0.13	56130.0	0.04	198935.9	0.01	999818.7	0.00
	16	8	146332.7	0.00	613541.1	0.00	1643568.6	0.00	10728199.9	0.00
		9	45153.1	0.01	207497.5	0.00	622796.8	0.00	3693356.1	0.00
		10	14134.2	0.05	76671.6	0.01	256860.2	0.00	1369569.6	0.00
		11	4889.9	0.13	32019.4	0.03	112627.0	0.01	557184.7	0.00
		12	2082.5	0.32	15686.4	0.09	55427.3	0.03	261976.6	0.01
		13	1236.0	0.57	9035.6	0.21	29396.8	0.07	140441.3	0.02
		14	1031.5	0.77	6140.5	0.38	18206.8	0.15	88410.3	0.05
		15	1085.0	0.87	4894.6	0.54	12505.5	0.25	62840.0	0.11
	20	8	99908.3	0.00	468968.4	0.00	1292360.9	0.00	8130494.3	0.00
		9	29895.2	0.02	159798.7	0.00	503454.8	0.00	2832660.7	0.00
		10	9268.8	0.05	60606.3	0.01	214923.5	0.00	1080437.5	0.00
		11	3107.0	0.13	25530.6	0.03	94343.0	0.01	442381.1	0.00
		12	1249.8	0.32	12386.8	0.08	44874.9	0.03	206143.4	0.01
		13	691.5	0.58	6806.6	0.19	22154.6	0.07	106374.1	0.02
		14	521.3	0.77	4226.8	0.36	12105.8	0.14	62201.9	0.04
		15	472.5	0.88	2913.9	0.53	6782.2	0.25	39088.7	0.10
		16	465.0	0.92	2218.6	0.65	4397.6	0.36	26998.0	0.19
		17	480.7	0.94	1815.7	0.73	2954.9	0.48	19736.7	0.31
		18	523.4	0.95	1609.7	0.78	2389.8	0.58	15836.2	0.42
		19	611.7	0.95	1568.1	0.81	2109.2	0.65	13447.3	0.51
	24	8	77006.4	0.00	396491.4	0.00	1116791.1	0.00	6819212.3	0.00
		9	22434.0	0.02	135141.4	0.00	437849.2	0.00	2376721.4	0.00
		10	6711.1	0.05	51516.0	0.01	186635.3	0.00	906467.2	0.00
		11	2227.6	0.14	22302.4	0.03	83831.8	0.01	381212.8	0.00
		12	892.5	0.32	11050.2	0.08	41005.2	0.03	183593.8	0.01
		13	479.8	0.57	6030.9	0.18	20205.1	0.06	95472.3	0.02
		14	349.6	0.77	3635.2	0.35	10761.0	0.13	55179.4	0.04
		15	301.1	0.88	2376.9	0.52	5728.7	0.23	33664.5	0.09
		16	279.5	0.93	1682.3	0.64	3413.0	0.35	21962.6	0.19
		17	271.4	0.94	1253.2	0.73	2041.8	0.47	15012.3	0.30
		18	266.8	0.95	990.7	0.78	1434.8	0.57	11022.0	0.41
		19	267.2	0.95	827.1	0.81	1034.7	0.66	8343.1	0.51
		20	272.9	0.96	724.7	0.83	875.9	0.72	6668.1	0.58
		21	286.4	0.96	672.2	0.85	774.7	0.77	5514.9	0.64
		22	314.5	0.96	659.1	0.86	760.8	0.80	4808.1	0.68
		23	369.4	0.96	704.7	0.87	806.4	0.83	4433.8	0.71

Supplementary Table 14: The table lists running time in seconds of using SubseqHash with different n and k in seeding step. The results are from the read mapping described in Section 3.5 on four datasets. For three real datasets, the running time includes seeding the reference genome and reads. For the simulated dataset, only the runing time of seeding simulated reads is shown.

Method	n	k	Running time			
			E.coli	S.cerevisiae	Simulation	D.melanogaster
SubseqHash	20	14	928.5	1407.5	626.5	11735.1
		15	871.0	1190.0	605.6	10569.8
		16	763.8	1155.1	534.0	9232.2
		17	629.4	947.5	445.9	7498.3
		18	465.4	608.0	322.1	5425.8
		19	282.6	379.7	200.4	3469.3
	25	16	1637.6	2380.5	1183.0	20081.4
		17	1513.3	2086.3	1036.2	19497.4
		18	1517.0	2238.1	965.2	17920.9
		19	1312.6	2065.3	969.4	16417.4
		20	1254.6	1618.4	750.2	14076.9
		21	1067.2	1571.4	691.5	12546.7
		22	817.6	1093.9	589.6	10067.0
		23	611.4	795.9	423.8	7238.8
		24	354.6	484.6	231.3	4391.4
	30	18	2399.5	3691.2	1753.5	30213.6
		19	2527.7	3543.9	1688.2	29115.3
		20	2189.5	3617.9	1670.5	28903.6
		21	2252.9	3475.1	1519.1	28929.3
		22	2013.6	3260.9	1527.1	25971.9
		23	1972.2	3003.3	1409.5	23954.2
		24	1797.5	2647.1	1259.1	21556.6
		25	1580.8	2359.0	1085.1	18851.0
		26	1358.1	1744.0	891.4	15741.0
		27	1083.2	1589.7	699.1	12598.6
		28	716.6	981.6	493.1	8682.9
		29	452.9	669.8	292.5	5336.1
	35	20	3519.9	4699.7	2359.5	40705.4
		21	3584.2	4987.5	2329.8	41909.9
		22	3460.6	5136.8	2416.0	41028.4
		23	3275.6	4975.9	2061.9	40058.6
		24	3271.5	4837.8	2180.5	38487.2
		25	3028.3	4628.2	2200.1	36857.1
		26	2943.1	4431.2	2056.0	34902.0
		27	2763.0	4067.4	1906.0	32540.8
		28	2440.2	3834.1	1698.9	28687.4
		29	2258.4	3336.0	1563.5	25317.5
		30	1933.3	2553.4	1357.1	22016.0
		31	1577.6	2420.9	1126.4	19122.1
		32	1228.2	1908.9	881.6	15005.9
		33	914.4	1268.5	627.5	10137.6
		34	531.5	699.8	364.1	6356.3

Supplementary Table 15: The table lists running time in seconds of Repeating SubseqHash for 10 times swith different n and k in seeding step. The results are from the read mapping described in Section 3.5 on four datasets. For three real datasets, the running time includes seeding the reference genome and reads. For the simulated dataset, only the runing time of seeding simulated reads is shown.

Method	n	k	Running time			
			E.coli	S.cerevisiae	Simulation	D.melanogaster
SubseqHash (repeating 10 times)	20	16	7486.8	11597.4	5386.0	91207.3
		17	6124.7	8987.7	4342.3	73738.6
		18	4558.6	6292.6	3248.7	55277.4
		19	2803.4	4214.5	2014.6	33758.9
	25	18	14738.1	22248.1	10415.3	180382.3
		19	13645.3	18480.0	9306.3	164136.3
		20	12362.2	16401.5	7950.2	145465.3
		21	10393.6	15228.0	7175.8	123831.6
		22	8397.2	11016.5	5814.2	100544.8
		23	5848.3	7963.3	4186.3	72325.6
		24	3599.0	5415.5	2453.8	44004.7
	30	20	22426.8	36140.3	15927.6	289132.2
		21	22115.1	34460.3	15676.9	278881.2
		22	21361.3	31523.1	15100.5	259393.1
		23	19485.9	29833.0	13390.4	238997.7
		24	17128.3	25469.4	12365.4	215576.2
		25	15760.8	22560.4	10568.2	184676.0
		26	13439.7	18642.5	8900.4	154548.4
		27	10344.1	15669.0	7052.6	124260.3
		28	7458.9	10939.4	4977.0	88156.2
		29	4481.3	6619.8	3008.4	53501.4
	35	22	34677.1	50838.0	23894.3	409452.7
		23	32323.6	48474.8	21739.4	399011.8
		24	32034.4	48269.2	22027.3	385128.4
		25	30408.2	44458.8	21553.5	369167.5
		26	28733.9	43702.3	20016.7	342461.4
		27	27489.7	40574.7	18732.0	322528.5
		28	23935.6	37552.3	17349.6	293984.9
		29	22515.6	31646.4	15572.4	263726.1
		30	18807.1	26865.1	13586.6	225657.3
		31	15734.5	23979.0	11201.1	190450.2
		32	12428.4	18957.6	8769.1	147341.1
		33	9060.8	13275.8	6134.4	106172.1
		34	5302.9	7044.6	3584.9	62701.8

Supplementary Table 16: The table lists running time in seconds of using kmer with different k in seeding step. The results are from the read mapping described in Section 3.5 on four datasets. For three real datasets, the running time includes seeding the reference genome and reads. For the simulated dataset, only the runing time of seeding simulated reads is shown.

Method	k	Running time			
		E.coli	S.cerevisiae	Simulation	D.melanogaster
kmer	9	6.9	9.6	4.1	81.2
	10	7.3	9.9	4.6	82.1
	11	6.2	9.9	4.5	83.3
	12	6.7	10.2	4.4	94.5
	13	6.4	10.6	4.6	96.4
	14	6.8	10.4	4.8	96.9
	15	6.9	10.4	4.5	97.8
	16	6.4	10.8	4.7	98.9
	17	6.3	10.5	4.8	99.1
	18	6.3	10.7	4.6	101.3
	19	6.8	10.6	4.8	102.5
	20	7.0	11.8	4.7	108.2
	21	6.9	11.6	4.8	104.7
	22	7.0	11.7	4.5	104.5
	23	7.5	10.1	4.9	106.8
	24	6.9	10.4	4.4	106.5
	25	6.8	9.6	4.3	105.3

Supplementary Table 17: The table lists running time in seconds of using minimizer with different n and k in seeding step. The results are from the read mapping described in Section 3.5 on four datasets. For three real datasets, the running time includes seeding the reference genome and reads. For the simulated dataset, only the runing time of seeding simulated reads is shown.

Method	n	k	Running time			
			E.coli	S.cerevisiae	Simulation	D.melanogaster
Minimizer	12	8	4.5	5.5	2.8	55.7
		9	4.3	5.9	2.9	58.2
		10	5.0	7.2	3.3	62.7
		11	5.5	8.8	3.8	85.7
	16	8	4.0	5.9	2.9	50.1
		9	4.0	6.0	2.4	50.7
		10	4.1	6.3	3.0	60.4
		11	4.1	6.3	2.8	64.7
		12	4.3	6.6	2.8	67.9
		13	4.3	6.7	2.9	69.7
		14	4.7	7.4	3.1	76.4
		15	5.8	8.8	3.6	90.9
	20	8	4.1	6.0	2.8	50.4
		9	4.1	5.6	2.5	56.2
		10	4.0	5.9	2.8	60.0
		11	3.5	6.0	2.7	58.9
		12	3.9	5.3	2.9	59.9
		13	3.9	5.4	2.6	60.9
		14	4.1	6.3	2.7	64.6
		15	4.1	6.6	2.8	65.9
		16	4.1	6.9	3.0	65.3
		17	4.3	7.1	3.1	68.2
		18	5.1	7.9	3.2	83.5
		19	5.6	9.2	3.9	101.1
	24	8	4.3	6.3	2.5	52.7
		9	4.2	6.3	2.7	60.6
		10	3.9	6.0	2.6	60.7
		11	4.1	5.9	2.8	62.0
		12	3.9	5.9	2.7	62.3
		13	3.7	5.4	2.7	64.0
		14	3.6	5.5	2.5	64.5
		15	3.8	6.1	2.5	65.5
		16	3.9	6.3	2.7	65.5
		17	3.8	6.0	2.8	66.9
		18	3.7	6.5	2.7	69.8
		19	4.3	6.2	3.0	72.7
		20	4.5	6.6	3.1	78.5
		21	4.5	6.9	3.1	81.5
		22	5.0	7.1	3.6	82.7
		23	5.7	8.6	3.9	97.6
